# Metixene is an incomplete autophagy inducer in preclinical models of metastatic cancer and brain metastases

**DOI:** 10.1172/JCI161142

**Published:** 2023-12-15

**Authors:** Jawad Fares, Edgar Petrosyan, Deepak Kanojia, Crismita Dmello, Alex Cordero, Joseph T. Duffy, Ragini Yeeravalli, Mayurbhai H. Sahani, Peng Zhang, Aida Rashidi, Victor A. Arrieta, Ilya Ulasov, Atique U. Ahmed, Jason Miska, Irina V. Balyasnikova, C. David James, Adam M. Sonabend, Amy B. Heimberger, Maciej S. Lesniak

**Affiliations:** 1 Department of Neurological Surgery, and; 2 Northwestern Medicine Malnati Brain Tumor Institute, Lurie Comprehensive Cancer Center, Feinberg School of Medicine, Northwestern University, Chicago, Illinois, USA.; 3 Dr. Vikram Sarabhai Institute of Cell and Molecular Biology, Faculty of Science, The Maharaja Sayajirao University of Baroda, Vadodara, Gujarat, India.

**Keywords:** Neuroscience, Oncology, Autophagy, Brain cancer, Breast cancer

## Abstract

A paucity of chemotherapeutic options for metastatic brain cancer limits patient survival and portends poor clinical outcomes. Using a CNS small-molecule inhibitor library of 320 agents known to be blood-brain barrier permeable and approved by the FDA, we interrogated breast cancer brain metastasis vulnerabilities to identify an effective agent. Metixene, an antiparkinsonian drug, was identified as a top therapeutic agent that was capable of decreasing cellular viability and inducing cell death across different metastatic breast cancer subtypes. This agent significantly reduced mammary tumor size in orthotopic xenograft assays and improved survival in an intracardiac model of multiorgan site metastases. Metixene further extended survival in mice bearing intracranial xenografts and in an intracarotid mouse model of multiple brain metastases. Functional analysis revealed that metixene induced incomplete autophagy through N-Myc downstream regulated 1 (NDRG1) phosphorylation, thereby leading to caspase-mediated apoptosis in both primary and brain-metastatic cells, regardless of cancer subtype or origin. CRISPR/Cas9 KO of *NDRG1* led to autophagy completion and reversal of the metixene apoptotic effect. Metixene is a promising therapeutic agent against metastatic brain cancer, with minimal reported side effects in humans, which merits consideration for clinical translation.

## Graphical abstract

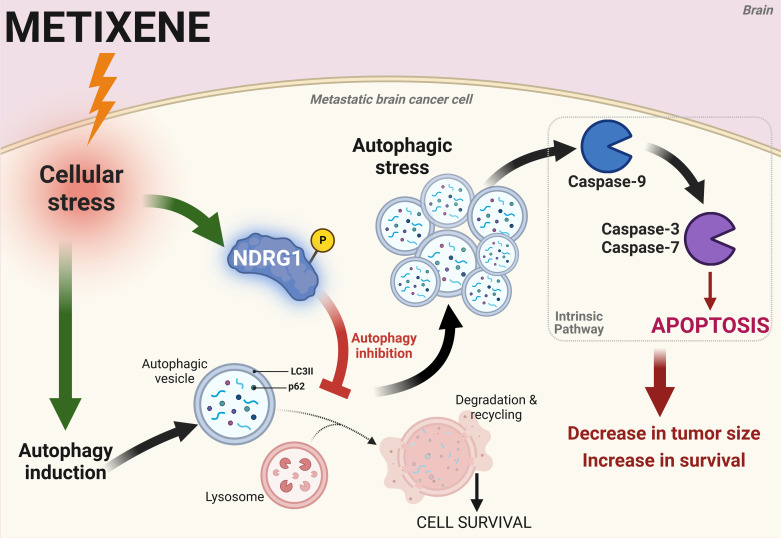

## Introduction

Metastasis is a hallmark of cancer that remains a primary cause of cancer-related deaths (1). Brain metastases are the most common type of CNS malignancies. They often manifest with neurological impairment that portends a poor quality of life and limits survival outcomes. It is estimated that 10%–30% of all patients with cancer will develop brain metastases at some point in their disease (2). The incidence of peripheral cancer spread to the CNS may be increasing, though, as a result of improved diagnostics and better control of extracranial disease through systemic therapies (3, 4).

Breast cancer is one of the major causes of brain metastases (5). It is the most common cancer among women, affecting 2.3 million women per year worldwide (6). It is also the most common cause of cancer-related deaths in women, with rates increasing in nearly every region globally (7). The incidence of brain metastases depends on the breast cancer molecular subtype, with human epidermal growth factor receptor 2–positive (HER2-positive) and triple-negative breast cancers having rates of brain metastases as high as 50% (8, 9). The introduction of the anti-HER2 monoclonal antibody trastuzumab for patients with HER2-positive breast cancer improved survival outcomes in systemic disease (10). Yet, HER2-positive breast cancers are at the greatest risk for intracranial-specific metastases. When metastatic cancer cells colonize the brain, they have been found to develop resistance to trastuzumab (11, 12). In addition, the poor penetration of trastuzumab and other systemically administered drugs across the blood-brain barrier (BBB) limits their effectiveness against breast cancer brain metastases (BCBM) (13–15).

A major limitation in treating patients with BCBM is the lack of clinical trials and new therapeutic options. With more than 10,000 registered clinical trials for breast cancer, less than 1% include patients with brain metastases (16). Moreover, of the trials that include patients with brain metastases, less than 15% are being completed, and less than 25% have published results (16). As such, novel, effective agents against the disease that can be swiftly translated to the clinic are urgently needed.

In this study, we conducted a CNS small-molecule inhibitor screen with agents that are known to be permeable through the BBB and have been approved by the FDA on BCBM cell lines that are sensitive and resistant to trastuzumab. We identified metixene, an antiparkinsonian drug, as a potent agent that induces caspase-mediated cell death in primary breast and brain-metastatic cancer cells. In vivo, metixene significantly decreased tumor size in primary breast cancer and significantly increased survival in multiple preclinical models of metastatic breast cancer and brain metastases (*P* < 0.05). Functional proteomics analysis highlighted signaling pathways that are involved in the cellular stress response and macroautophagy. Macroautophagy involves the formation of double-membrane autophagosomes that bind to lysosomes to form single-membrane autolysosomes, where cargo can be degraded through lysosomal hydrolases (17). The accumulation of such autophagic structures can lead to autophagic stress that induces caspase-mediated apoptosis (18).Further exploration proved that metixene induced N-Myc downstream regulated 1–mediated (NDRG1-mediated) incomplete autophagy, in which accumulated autophagic vesicles did not degrade, triggering caspase-mediated apoptosis in HER2-positive and triple-negative metastatic breast cancer and brain metastases.

## Results

### A CNS small-molecule inhibitor screen identifies agents with anticancer effects in BCBM.

**Figure 1:**
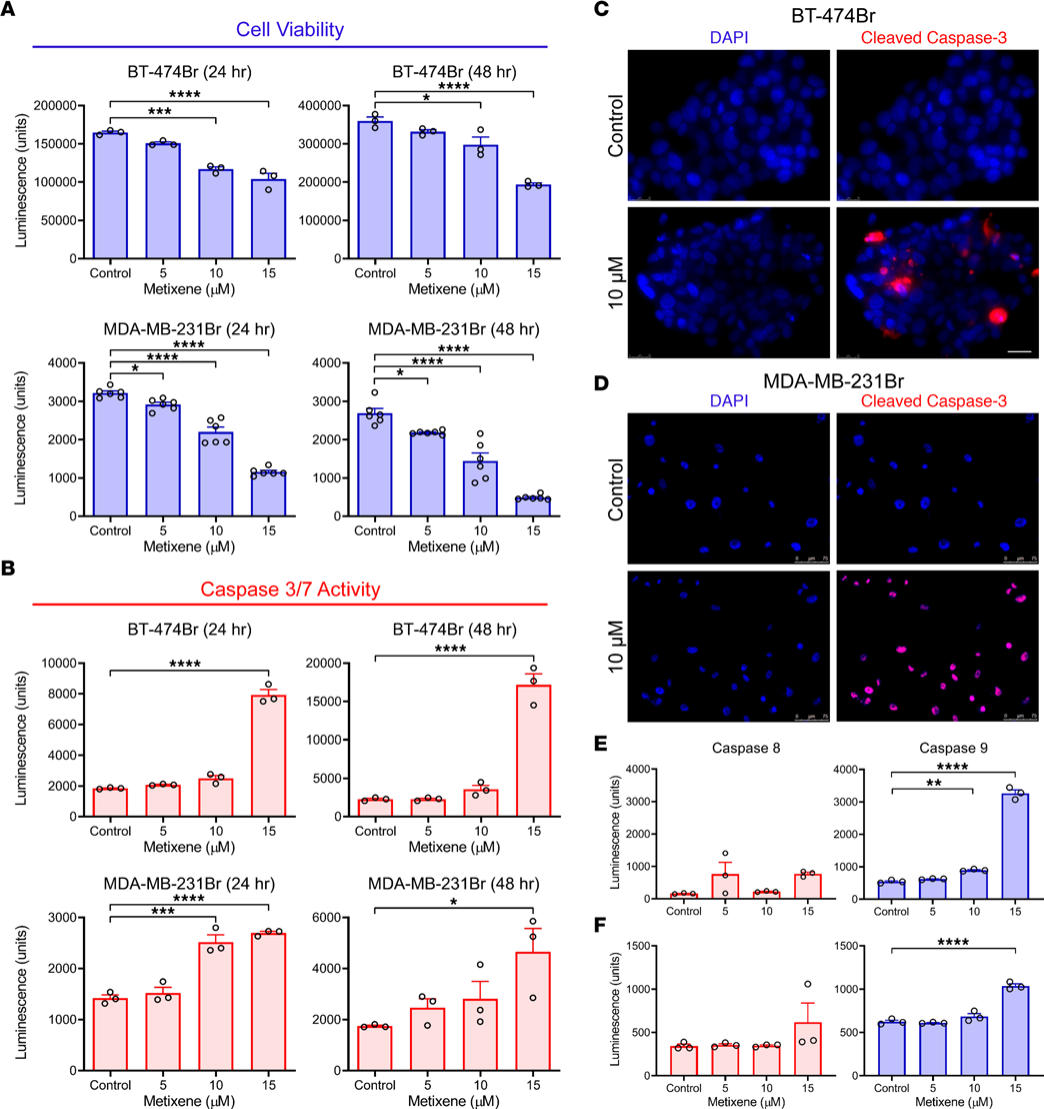
Metixene induces apoptotic cell death in brain-metastatic breast cancer cells. (**A**) Viability of BT-474Br and MDA-MB-231Br cells under treatment at different concentrations of metixene for 24 hours and 48 hours. (**B**) Caspase-3/-7 activity in BT-474Br and MDA-MB-231Br cells under treatment at different concentrations of metixene for 24 hours and 48 hours. (**C**) Immunofluorescence staining for cleaved caspase-3 after metixene treatment of BT-474Br cells for 2 days. Scale bars: 25 μm. (**D**) Immunofluorescence staining for cleaved caspase-3 after metixene treatment in MDA-MB-231Br cells for 2 days. Scale bars: 75 μm. (**E**) Caspase-8/-9 activity in BT-474Br cells under treatment at different concentrations of metixene for 24 hours. (**F**) Caspase-8/-9 activity in MDA-MB-231Br cells under treatment at different concentrations of metixene for 24 hours. The results in **A**, **B**, **E**, and **F** are representative of 3 or more technical replicates, and bar graph data show the mean ± SEM. *P < 0.05, **P < 0.01, ***P < 0.001, and ****P < 0.0001, by 1-way ANOVA with Dunnett’s post hoc test.

A blinded screen was performed to identify potential hits for therapeutic intervention against BCBM using the Prestwick CNS Drug Library consisting of 320 structurally diverse drugs that are FDA approved, BBB permeable, and known for their pharmacological effects in the CNS. The first screen was on BT-474Br, a HER2-positive BCBM cell line, to prioritize cytotoxic agents at a standard concentration of 25 μM. The screen yielded 5 different therapeutic agents that caused a greater than 95% reduction in cellular viability in vitro (Table 1). As HER2-positive BCBMs are believed to be resistant to trastuzumab (19), we conducted the second screen on the HCC1954 metastatic breast cancer cell line that is inherently resistant to therapy (20) (Table 1). This revealed metixene, an antiparkinsonian agent, as a top candidate in both screens. To evaluate the effect of metixene on a panel of various metastatic breast cancer cell lines, the IC_50_ was determined in 2 HER2-positive cell lines (BT-474Br and HCC1954) and 5 triple-negative breast cancer cell lines (MDA-MB-231Br, HCC1806, HS578T, HCC3153, and SUM159) after 72 hours of treatment. We found that metixene was a potent inhibitor of BCBM regardless of breast cancer subtype, with an IC_50_ ranging from 9.7 μM to 31.8 μM (Table 2 and Supplemental Figure 1; supplemental material available online with this article; https://doi.org/10.1172/JCI161142DS1). Following the determination of an appropriate range for treatment of BCBM cells, we evaluated the anticancer activity of metixene in the brain-metastatic HER2-positive BT-474Br and the triple-negative MDA-MB-231Br cell lines using increasing concentrations of the drug at different time points. After both 24 and 48 hours of treatment, a dose response was evident in cellular viability assays for both cell lines (Figure 1A).

### Metixene induces caspase-mediated apoptosis in cancer cells.

Flow cytometry results indicated that metixene was inducing cell death in cancer cells (Supplemental Figure 2). To clarify the mechanism of cell death, we assessed the caspase cascade. A caspase-3/-7 assay was performed to check for caspase-mediated apoptosis, which showed a significant increase in activity that was dependent on dose and time in the BT-474Br and MDA-MB-231Br cells (Figure 1B). We performed immunofluorescence studies to confirm that metixene induced caspase-3 cleavage in the 2 BCBM cell lines (Figure 1, C and D). The caspase-8 and -9 assays were also used to measure the effect of metixene in BCBM cells. After 24 hours, we found that caspase-9 activity was significantly elevated at 10 μM in the BT-474Br cells (*P* = 0.0055), and at 15 μM in the BT-474Br cells (*P* < 0.0001) and the MDA-MB-231Br cells (*P* < 0.0001) (Figure 1, E and F). These data indicate that the intrinsic pathway of apoptosis was activated as a result of metixene treatment.

### Metixene decreases tumor size and improves survival in murine models of established metastatic breast cancer.

**Figure 2:**
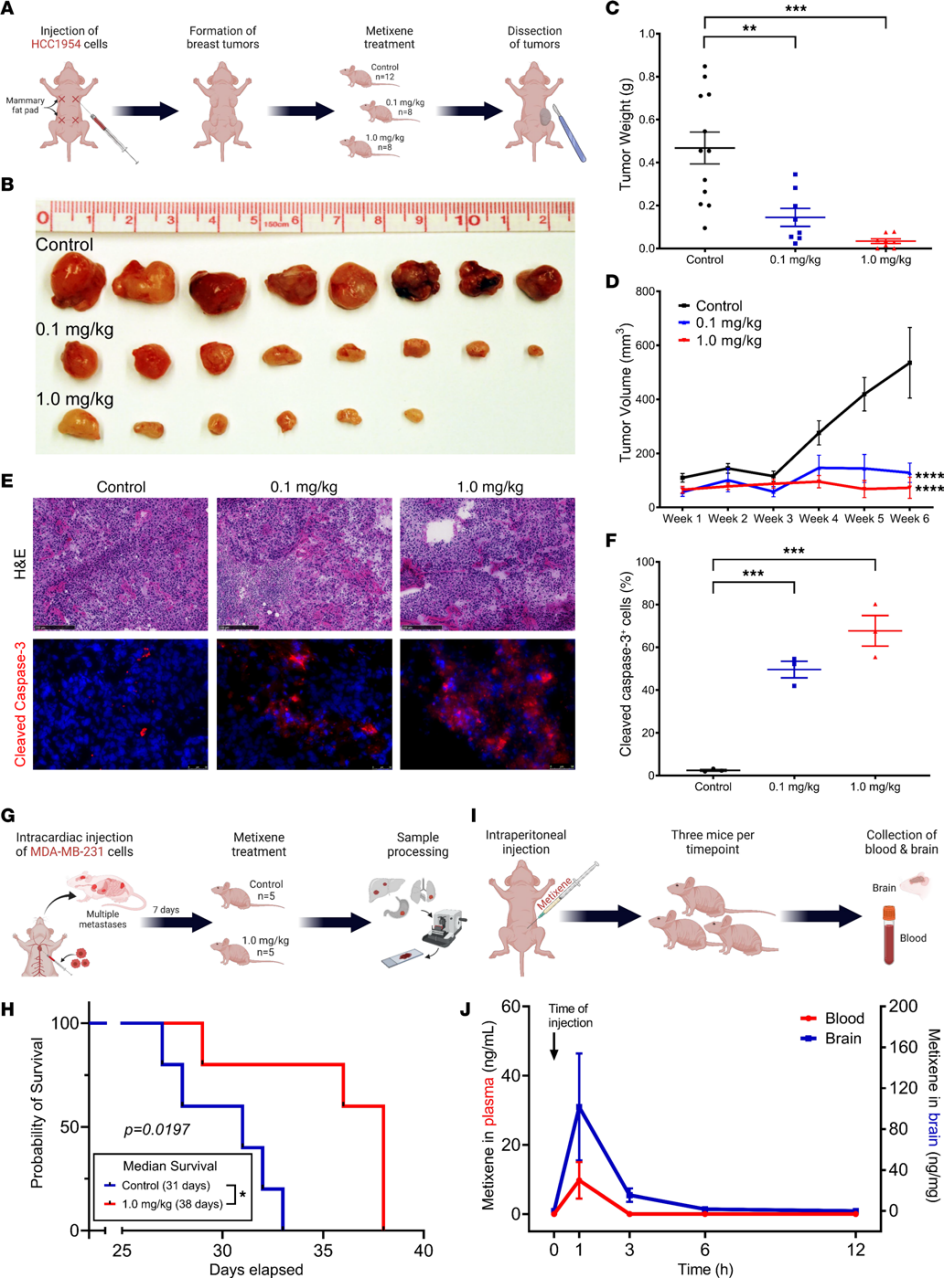
Metixene’s anticancer efficacy decreases the size of mammary fat pad tumors and improves survival in a murine model of metastatic breast cancer. (**A**) Experimental setup of mammary fat pad injections with HCC1954 cells and subsequent treatment with metixene. (**B**) Metixene treatment at 0.1 mg/kg and 1.0 mg/kg decreased tumor sizes in comparison with controls. (**C**) Tumor weight at 0.1 mg/kg and 1.0 mg/kg of metixene treatment. (**D**) Tumor volume at 0.1 mg/kg and 1.0 mg/kg of metixene treatment. (**E**) H&E (scale bar: 250 μm) and cleaved caspase-3 (scale bar: 50 μm) staining of tumors treated with control, 0.1 mg/kg, and 1.0 mg/kg metixene. Red color indicates cleaved caspase-3; blue color represents DAPI. (**F**) Percentage of cleaved caspase-3–positive cells (*n* = 3 per group). (**G**) Experimental setup for intracardiac injections of MDA-MB-231 cells and subsequent treatment with metixene. (**H**) Kaplan-Meier curve showing survival of metixene-treated mice versus control mice following intracardiac injection of MDA-MB-231 cells. (**I**) Experimental setup to determine the bioavailability of metixene in blood and brain tissue. (**J**) Metixene levels in plasma and brain tissue across time after intraperitoneal administration. **P < 0.01, ***P < 0.001, and ****P < 0.0001, by 1-way ANOVA with Dunnett’s post hoc test (**C** and **F**) 2-way ANOVA with Tukey’s post hoc test (**D**). Survival curves were compared using a log-rank test.

To ascertain whether metixene could exert in vivo therapeutic activity, HCC1954 cells were orthotopically implanted into the mammary fat pads of nude mice. When the tumors reached 5 mm in size, the mice were randomized into 3 groups: control (25% captisol, *n* = 12), metixene (0.1 mg/kg, *n* = 8), and metixene (1.0 mg/kg, *n* = 8), and were treated intraperitoneally 3 times per week (Figure 2A). After 6 weeks, the fat pad tumors were collected and analyzed (Figure 2B). Tumor weights were significantly decreased with metixene treatment at 0.1 mg/kg (*P* < 0.0001) and 1.0 mg/kg (*P* < 0.0001) (Figure 2C). Tumor volumes were also significantly reduced with metixene treatment at 0.1 mg/kg (*P* = 0.0043) and 1.0 mg/kg (*P* = 0.0004) (Figure 2D). Cleaved caspase-3 staining of tumor sections showed a significant increase in the percentage of cleaved caspase-3–positive cells in the metixene-treated groups at 0.1 mg/kg (*P* = 0.001) and 1.0 mg/kg (*P* = 0.0002) (Figure 2, E and F).

In an experimental model of metastatic breast cancer involving multiple organ sites, triple-negative MDA-MB-231 cells were intracardially injected into the left ventricle of immunodeficient mice. Following a 7-day-interval post-tumor cell inoculation, the mice were randomly assigned to 2 distinct cohorts: a control group (25% captisol, *n* = 5) and a metixene treatment group (1.0 mg/kg, *n* = 5). The treatment regimen involved intraperitoneal administration 3 times per week (Figure 2G). While the control mice had a median survival of 31 days, the mice receiving metixene had a significantly extended median survival of 38 days (*P* = 0.0197) (Figure 2H). Histological examination of both control and metixene-treated mice revealed a reduction in tumor burden within the lungs and large intestines of mice in the metixene-treated group. Furthermore, we observed no discernible tumor formation in the stomach or liver of these treated mice (Supplemental Figure 3).

To assess the pharmacokinetics and bioavailability of metixene, our study aimed to quantitatively analyze metixene concentrations in both blood plasma and brain tissue. Metixene was administered via intraperitoneal injection, and the mice were randomly divided into 5 distinct groups, each corresponding to a specific time point after administration. At each designated time point, comprising a cohort of 3 mice, the animals were humanely sacrificed. Subsequently, 500 μL blood was collected from each mouse, and their brain tissues were harvested for analytical purposes (Figure 2I). The analysis revealed a peak concentration of metixene in both blood plasma and brain tissue occurring approximately 1 hour after injection, reaching an average of 9.7 ng/mL in the plasma and 101.6 ng/mg in the brain tissue. Metixene was completely cleared from the plasma within 3 hours of administration and from brain tissue within a 12-hour time frame (Figure 2J).

### Metixene improves in vivo survival in preclinical models of metastatic brain cancer.

**Figure 3:**
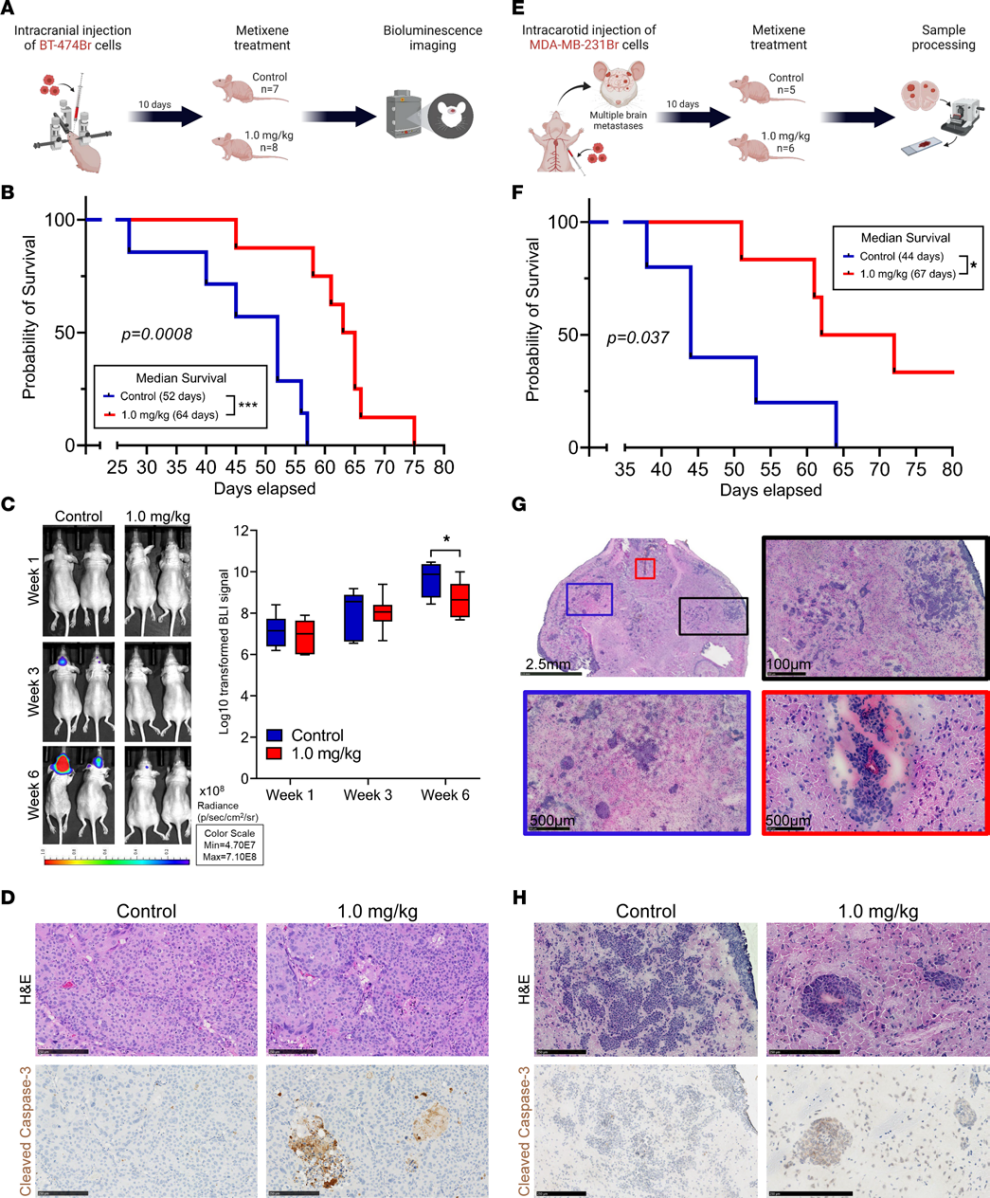
Metixene improves survival in preclinical models of metastatic brain cancer. (**A**) Experimental setup for stereotactic intracranial injection of BT-474Br cells into the brains of nude mice and subsequent treatment with metixene. (**B**) Kaplan-Meier curve showing survival of metixene-treated mice versus survival of control mice upon intracranial injection of BT-474Br cells. (**C**) Bioluminescence imaging of the 2 groups (control vs. treated) across time. (**D**) Immunohistochemical staining for cleaved caspase-3 in brain samples from control and metixene-treated mice bearing BT-474Br tumors (quantification of the stained area is shown in Supplemental Figure 4A). Scale bars: 250 μm. (**E**) Experimental setup for intracarotid artery injection of MDA-MB-231Br cells into nude mice and subsequent treatment with metixene. (**F**) Kaplan-Meier curve showing survival of metixene-treated mice versus controls upon intracarotid injection of MDA-MB-231Br cells. (**G**) Histological brain sections upon death of the control mice confirmed the growth of brain tumors as a result of intracarotid injection of MDA-MB-231Br cells. H&E staining of mouse brain shows metastatic tumors (black-framed box), the formation of multiple micrometastases (blue-framed box), and vascular co-option (red-framed box). Scale bars: 2.5 mm; 100 μm, and 500 μm. (**H**) Immunohistochemical staining for cleaved caspase-3 in brain samples from control and metixene-treated mice bearing MDA-MB-231Br tumors (quantification of the stained area is shown in Supplemental Figure 4B). Scale bars: 250 μm. Survival curves were compared using a log-rank test. The linear mixed model was fitted for **C**, and the differences between the 2 groups for each time point were calculated using the least-squares means method and adjusted by Šidák’s method. *P < 0.05 and ***P < 0.001.

To ascertain whether metixene exerts therapeutic activity against breast cancer in the brain, we stereotactically implanted HER2-positive BT-474Br cells into the brains of nude mice. After 10 days, the mice were randomized into 2 groups: control (25% captisol, *n* = 7) and metixene (1.0 mg/kg, *n* = 8), and were treated intraperitoneally 3 times a week (Figure 3A). The metixene-treated mice had a 23% increase in median survival, with a median survival period of 64 days, in contrast to the median survival of 52 days for the control mice (*P* = 0.0008) (Figure 3B). This finding was consistent with the measurement of the luciferase signal in the brain when the BT-474Br cells were fluorescently labeled and treated with metixene relative to controls after 6 weeks of treatment (*P* = 0.039) (Figure 3C). Immunohistochemical staining of the brains of treated mice confirmed that metixene significantly induced caspase-3 cleavage in the established HER2-positive BCBMs in vivo (Figure 3D and Supplemental Figure 4A).

In a multiple brain metastases model, we injected triple-negative MDA-MB-231Br cells into the carotid artery of nude mice. Ten days after injection of tumor cells, the mice were randomized into 2 groups: control (25% captisol, *n* = 5) and metixene (1.0 mg/kg, *n* = 6) and were treated intraperitoneally 3 times per week (Figure 3E). In comparison with the control mice, which had a median survival of 44 days, the metixene-treated mice had a median survival of 67 days. Therefore, metixene significantly increased survival by 52% in comparison with controls (*P* = 0.037) (Figure 3F). Histological examination of the brains harvested from control-treated mice showed multiple metastases, vascular co-option, and the formation of micrometastases (Figure 3G). Histological comparison of brains of control and metixene treated mice showed a decreased tumor burden in the metixene-treated mice (Supplemental Figure 5). Immunohistochemical staining showed that cleaved caspase-3 was significantly higher in the BCBM tissue of the metixene-treated mice in vivo (Figure 3H and Supplemental Figure 4B).

Toxicity studies conducted by treating nontumor-bearing nude mice (*n* = 4) with the same metixene regimen (1 mg/kg; intraperitoneally; 3 times/week) for more than 3 months confirmed the absence of toxic or adverse effects when compared with untreated mice (*n* = 4). The difference in body weight of treated and control mice was not significant (Supplemental Figure 6A). Evaluation of organ tissue, such as the brain, heart, lung, liver, pancreas, kidneys, spleen, stomach, and intestines, revealed no differences in weight or anatomical architecture between the treated and untreated mice (Supplemental Figure 6, B and C).

### Metixene induces cellular stress and activates macroautophagy signaling pathways.

**Figure 4:**
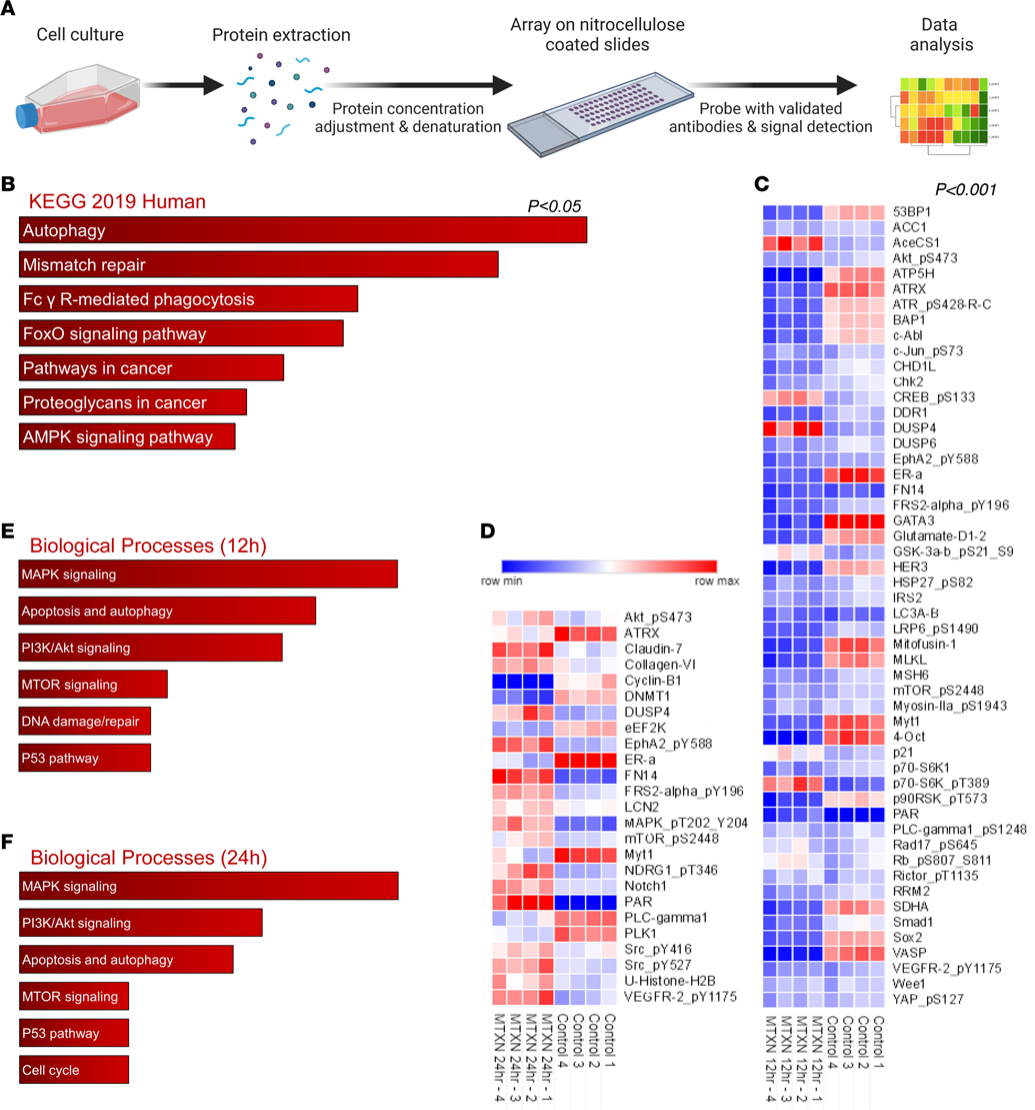
RPPA highlights autophagy signaling in breast cancer brain-metastatic cells. (**A**) Experimental setup for RPPA of BT-474Br cells subjected to metixene treatment for 12 hours and 24 hours, respectively. (**B**) Pathway analysis of changes in protein phosphorylation that were significant, with a *P* value of less than 0.05, using the Kyoto Encyclopedia of Genes and Genomes (KEGG) 2019 human database. (**C**) Heatmap of protein phosphorylation changes that were significant, with a *P* value of less than 0.001 at 12 hours. (**D**) Heatmap of protein phosphorylation changes that were significant, with a *P* value of less than 0.001 at 24 hours. (**E**) Biological processes that were significantly activated after 12 hours of metixene treatment. (**F**) Biological processes that were significantly activated after 24 hours of metixene treatment. Statistical analysis was performed using a 2-tailed Student’s *t* test to compare the means of protein/phosphorylation expression between control samples and metixene-treated samples.

To clarify the mechanism of action of metixene, an agent with known antimuscarinic and antihistaminic properties (21), the expression of muscarinic and histamine receptors on the breast cancer cell lines was determined and correlated with the IC_50_. RNA levels of either muscarinic (M1–M5) or histamine (H3) receptors did not correlate with the induced cytotoxicity of metixene (Supplemental Figure 7), indicating that the anticancer activity of metixene was not a function of muscarinic or histaminic receptors. As such, to identify in an unbiased fashion the underlying mechanism of action of metixene, we treated BT-474Br cells with metixene and conducted a reverse-phase protein array (RPPA) analysis to assess the changes in signaling pathways (Figure 4A). Four replicates for each condition (control, 12 hours, and 24 hours) were analyzed and revealed that autophagy is a primary biological mechanism regulated by metixene (*P* < 0.05) (Figure 4B and Supplemental Figure 8). Among the proteins that exhibited significant changes (with *P* < 0.001) in expression or phosphorylation, 52 and 26 proteins, respectively were significantly altered (Figure 4, C and D). These proteins implicated the signaling pathways for MAPK, PI3K/Akt, apoptosis and autophagy, and mTOR that are involved in the process of macroautophagy. Other proteins implicated in DNA damage and repair, P53, and cell-cycle control pathways were also affected, indicating cellular stress induction as a result of metixene treatment (Figure 4, E and F, and Supplemental Tables 1 and 2).

**Figure 5:**
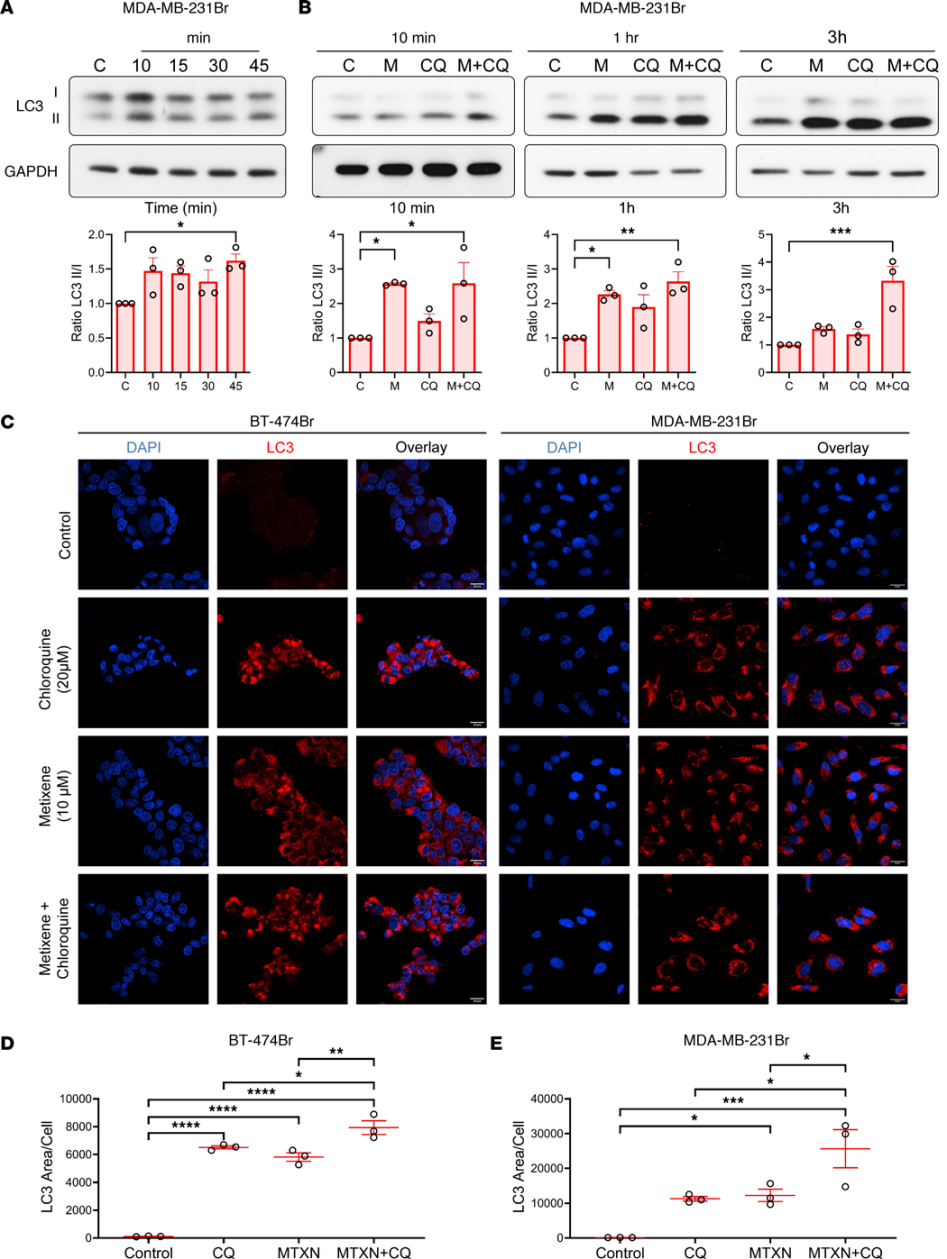
Metixene induces autophagy signaling in metastatic brain cancer cells. (**A**) Western blot and analysis of LC3 protein expression in MDA-MB-231Br cells following metixene treatment (10 μM) at the specified time points. (**B**) Western blot and analysis of autophagy flux protein expression in MDA-MB-231Br cells treated with metixene (M) (10 μM) and/or chloroquine (CQ) (20 μM) at different time points. (**C**) Representative LC3 puncta immunofluorescence in BT-474Br and MDA-MB-231Br cells under metixene treatment (10 μM) for 48 hours, chloroquine (20 μM) for 24 hours, and the combination of both metixene and chloroquine. Scale bars: 20 μm. (**D**) Quantification of the area of LC3 puncta per cell in BT-474Br cells upon treatment with control, metixene (MTXN), chloroquine, or the combination of metixene and chloroquine. (**E**) Quantification of the area of LC3 puncta per cell in MDA-MB-231Br cells upon treatment with control, metixene, chloroquine, or metixene plus chloroquine. Results are representative of 3 independent experiments. *P < 0.05, **P < 0.01, ***P < 0.001, and ****P < 0.0001, by 1-way ANOVA with Dunnett’s or Tukey’s post hoc test.

Next, we treated MDA-MB-231Br cells with metixene for different durations to further characterize the mechanisms of autophagic signaling in brain-metastatic cells. A strategy for monitoring autophagy is the detection of LC3 conversion (LC3I to LC3II) by immunoblotting, since the amount of LC3II can be associated with the number of autophagosomes (22, 23). Conversion of LC3I to LC3II was shown to increase as early as 10 minutes after metixene treatment (Figure 5A). Thereafter, autophagy flux changes were tested 10 minutes, 1 hour, and 3 hours after treatment. We compared LC3II levels in untreated control cells with cells treated with chloroquine (20 μM), an autophagy flux inducer, metixene (10 μM), or the combination of metixene and chloroquine. Cells treated with the combination of metixene and chloroquine had higher LC3II levels than levels detected in the untreated cells or in the cells treated with either agent alone (Figure 5B). This finding was further confirmed with immunofluorescence staining for LC3 (Figure 5C). Quantification of the area of LC3 puncta per cell showed a significant increase (*P* < 0.05) upon the addition of chloroquine to metixene in both BT-474Br and MDA-MB-231Br cells (Figure 5, D and E). Immunofluorescence staining for LC3 in metixene-treated cells in a dose-dependent manner also showed a significant increase in the area of LC3 puncta per cell as the dose was increased in 2 BCBM cell lines (Supplemental Figure 9). The addition of wortmannin, a PI3K inhibitor and an early inhibitor of autophagy flux (24), to metixene led to a decrease in LC3II expression (Supplemental Figure 10). Altogether, these data indicate that metixene induced autophagy signaling in cancer cells.

### Metixene induces incomplete autophagy in cancer cells.

**Figure 6:**
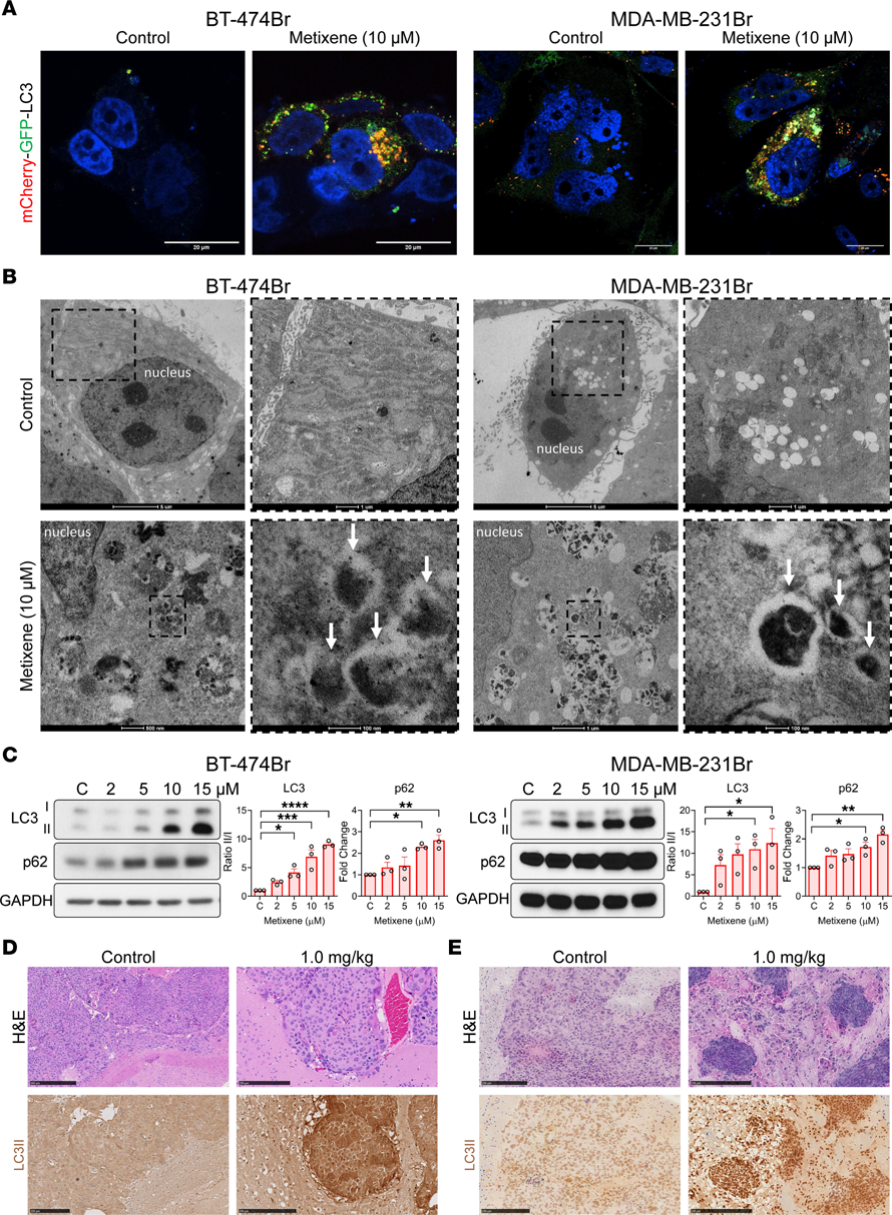
Metixene induces incomplete autophagy in metastatic brain cancer cells. (**A**) Control and metixene-treated BT-474Br and MDA-MB-231Br cells expressing mCherry-GFP-LC3. Autophagosomes display both GFP and mCherry fluorescence, appearing yellow/green. Autolysosomes showcase mCherry fluorescence only, as GFP was denatured by acidic lysosomes, appearing red. Scale bars: 20 μm. (**B**) Electron microscopic images of metixene-treated BT-474Br and MDA-MB-231Br cells (white arrows show double-membraned autophagosomes). Scale bars: 5 μm (top left), 1 μm (enlarged insets, top right) and 500 nm (bottom left), 100 nm (enlarged insets, bottom right). (**C**) Western blots and analysis of LC3 and p62 protein expression in BT-474Br and MDA-MB-231Br cells treated with increasing concentrations of metixene (μM) for 48 hours. (**D**) Immunohistochemical staining for LC3B in brain samples of control and metixene-treated mice bearing BT-474Br tumors (quantification of the stained area is shown in Supplemental Figure 12A). (**E**) Immunohistochemical staining for LC3B in brain samples from control and metixene-treated mice bearing MDA-MB-231Br tumors (quantification of the stained area is shown in Supplemental Figure 12B). Scale bars: 250 μm. Results are representative of 3 independent experiments. *P < 0.05, **P < 0.01, ***P < 0.001, and ****P < 0.0001, by 1-way ANOVA with Dunnett’s post hoc test.

Using a system in which LC3, the marker of autophagosomes, is fused to both GFP and mCherry, we found that metixene-treated BT-474Br and MDA-MB-231 cells contained numerous autophagosomes (yellow/green), which do not bind to lysosomes that degrade their contents (red) (Figure 6A). Electron microscopic analysis of metixene-treated cells at 10 μM showed accumulation of double-membraned autophagic vesicles in the cytoplasm of BT-474Br and MDA-MB-231Br cells (Figure 6B). Dose-dependent treatment of the 2 cell lines showed a significant increase in LC3I/-II levels in BT-474Br (*P* < 0.0001) and MDA-MB-231Br (*P* = 0.0487) cells. Similar results were confirmed in primary breast cancer cell lines (Supplemental Figure 11). Furthermore, the levels of p62, an autophagy cargo protein that binds other proteins for selective autophagy, increased significantly in BT-474Br (*P* = 0.0033) and MDA-MB-231Br (*P* = 0.0043) cells, indicating that the content of autophagic vesicles was not being degraded and that autophagy was incomplete (Figure 6C). Immunohistochemical staining of brain samples from control and metixene-treated mice showed that LC3B was significantly higher in metixene-treated samples in both BT-474Br intracranial models and MDA-MB-231Br intracarotid injection models (Figure 6, D and E, and Supplemental Figure 12).

### NDRG1 regulates metixene-induced incomplete autophagy and caspase-mediated apoptosis.

**Figure 7:**
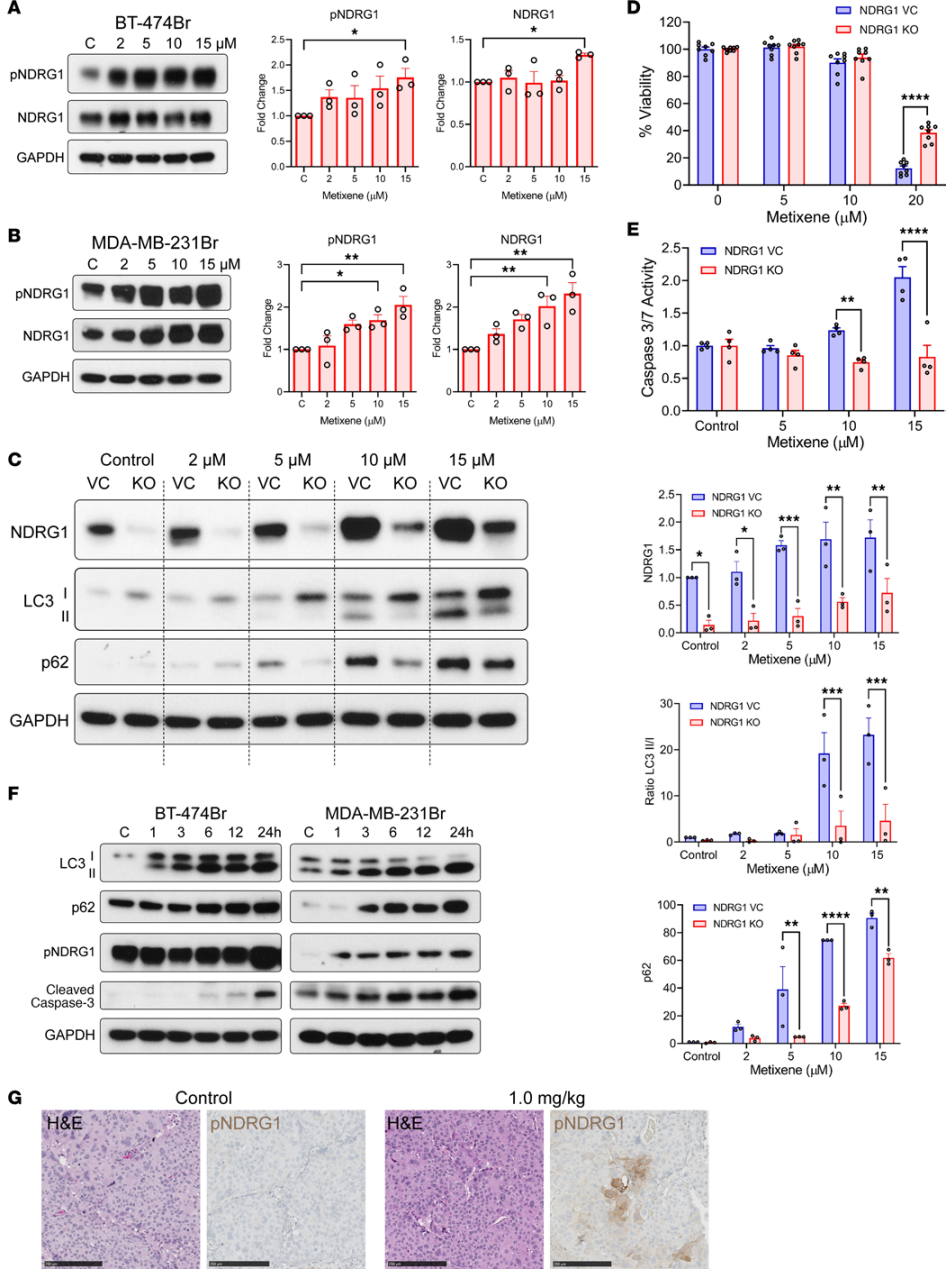
NDRG1-mediated incomplete autophagy induces apoptotic cell death in metastatic brain cancer cells. (**A**) Western blots and analysis p-NDRG1 and NDRG1 protein expression in BT-474Br cells treated with increasing concentrations of metixene (μM). (**B**) Western blots and analysis of p-NDRG1 and NDRG1 protein expression in MDA-MB-231Br cells treated with increasing concentrations of metixene (μM). (**C**) Western blots and analysis of protein expression in NDRG1-KO versus VC-transfected MDA-MB-231Br cells treated with increasing concentrations of metixene (μM). (**D**) Viability of NDRG1-KO versus VC-transfected MDA-MB-231Br cells under treatment with different concentrations of metixene for 24 hours. (**E**) Caspase-3/-7 activity in NDRG1-KO versus VC-transfected MDA-MB-231Br cells under treatment with different concentrations of metixene for 24 hours. (**F**) Protein expression of LC3, p62, p-NDRG1, and cleaved caspase-3 in BT-474Br and MDA-MB-231Br cells treated with metixene in a time-dependent manner. (**G**) Immunohistochemical staining for p-NDRG1 in brain samples from control and metixene-treated mice bearing BT-474Br tumors (quantification of the stained area is shown in Supplemental Figure 13). Scale bars: 250 μm. Results in **A**–**C** are representative of 3 independent experiments. Results in **D** are representative of 8 technical replicates. Results in **E** are representative of 4 technical replicates. *P < 0.05, **P < 0.01, ***P < 0.001, and ****P < 0.0001, by 2-way ANOVA with Šidák’s post hoc test.

Upon further analysis of the RPPA data, we noted that the phosphorylation of NDRG1 was relevant in 4 major pathways related to autophagy after 24 hours of metixene treatment. The expression of NDRG1 and its phosphorylated form, p-NDRG1, increased significantly in a dose-dependent manner in both BT-474Br and MDA-MB-231Br cells after treatment with metixene (Figure 7, A and B). To confirm the role of NDRG1 in autophagy, we generated NDRG1-KO cells using CRISPR/Cas9 in MDA-MB-231Br cells. The cells were then treated with metixene in a dose-dependent manner and compared with cells transfected with a vector control (VC). Western blots showed that LC3II levels decreased significantly in NDRG1-KO cells at 10 μM (*P* = 0.0009) and 15 μM (*P* = 0.0001). Furthermore, the significant decrease in p62 levels in NDRG1-KO cells at 5 μM (*P* = 0.0014), 10 μM (*P* < 0.0001), and 15 μM (*P* = 0.0073) indicated that autophagy was being completed in comparison with NDRG1 VC cells (Figure 7C). This showed that NDRG1 expression and phosphorylation regulated metixene-induced incomplete autophagy.

We then checked whether NDRG1 is further involved in the apoptotic cell death induced by metixene. We conducted a cell viability assay to check the effect of NDRG1 KO versus VC upon dose-dependent treatment with metixene. The results showed that NDRG1-KO cells were significantly more viable than control cells at 20 μM (*P* < 0.0001) (Figure 7D). A caspase-3/-7 assay further confirmed that apoptosis was significantly reduced in the NDRG1-KO cells at 10 μM (*P* = 0.0078) and 15 μM (*P* < 0.0001) (Figure 7E). To check the cascade of events upon metixene administration, we treated BT-474Br and MDA-MB-231Br cells in a time-dependent manner. Incomplete autophagy, as indicated by LC3I-to-LC3II conversion and accumulation of p62, preceded NDRG1 phosphorylation and eventual caspase-3 cleavage (Figure 7F). Altogether, this showed that metixene induced incomplete autophagy and intrinsic apoptosis through NDRG1 expression and phosphorylation. Upon NDRG1 KO, autophagy was completed, and the caspase-mediated pathway of apoptosis was not activated. Immunohistochemical staining for p-NDRG1 in murine brain samples showed that metixene treatment led to significantly higher expression of p-NDRG1 than in control samples (Figure 7G and Supplemental Figure 13). This mechanism of action was consistent in other types of metastatic brain cancer, such as lung cancer and melanoma (Supplemental Figure 14).

NDRG1, a marker of the cellular stress response, is known to be induced under conditions of cellular stress (25), including DNA damage. The other signaling pathways underscored by the RPPA analysis suggested a complex interplay between autophagy, DNA damage, and cell death. The downregulation of DNA damage repair and checkpoint proteins, such as ATRX, ATR, BAP1, and 53BP1, may exacerbate cellular stress induced by metixene, upregulating NDRG1 in the process and leading to incomplete autophagy. The accumulation of damaged DNA and organelles, in turn, may lead to cell-cycle arrest or apoptosis. These mechanisms can also affect cancer stem cells, as they are particularly sensitive to genomic instability and cellular stress (26). We found that stem cell markers, such as SOX2 and OCT4, were downregulated after metixene treatment (Supplemental Figure 15).

## Discussion

The current management of patients with metastatic brain cancer typically includes resection and/or radiation, with a median survival of less than 14 months (27). The development of novel therapeutics for patients with metastatic cancer that also have brain metastases is hindered by the slow pace of drug development, expense, and high attrition rates during clinical trials (28). Drug repositioning or repurposing in cancer is a strategy that aims to reuse existing medical agents developed for other diseases as anticancer treatments (29–31). The Repurposing Drugs in Oncology project was launched in 2014 to reposition reputable and well-characterized drugs as new agents in oncology based on evidence from published literature (32). In brain metastases, novel anticancer therapies must have the ability to cross the BBB and to instigate cytotoxic effects against metastatic cancer cells that are often resistant to standard therapies. We conducted a screening on trastuzumab-sensitive and -resistant brain-metastatic cells using a library of agents that are known to cross the BBB, which identified metixene as a translational candidate. The dose at which the survival of mice harboring established brain metastases was shown to be significant was 1 mg/kg given 3 times weekly. In humans, metixene can be administered at a starting dose of 2.5 mg 3 times a day and then increased gradually depending on the clinical response to a total of 15–60 mg daily in divided doses (33). The human equivalent dose calculation based on body surface area would be 0.0813 mg/kg (34). In a person who weighs between 50 and 60 kg, a metixene dose of 4–5 mg/day should be tolerable and realistic. This may allow a truncated phase 1 component, since metixene usually has mild antimuscarinic side effects (21).

Repositioning of antiparkinsonian drugs has shown to be effective against cancer metastasis. Specifically, benztropine, a second-line drug for the treatment of Parkinson’s disease, significantly inhibited in vivo tumor growth, reduced the number of circulating tumor cells, and decreased the rate of metastasis in a tumor allograft model in mice (35). Carbidopa, another antiparkinsonian medication, has also been shown to be effective against pancreatic cancer by the inhibition of indoleamine-2,3-dioxygenase-1 and the potentiation of aryl hydrocarbon receptor signaling (36). Epidemiological studies have shown that the relative risk of cancer development among patients treated for Parkinson’s disease was less than half that in the healthy population (37, 38). A systematic review and meta-analysis of the literature exploring cancer risk among patients with Parkinson’s disease further showed that the aggregate risk for cancer in patients with Parkinson’s disease was 0.73 (39).

Metixene was found to be a modulator of autophagy signaling in metastatic breast cancer and brain metastases. Recent genetic investigations and emerging functional research demonstrate the existence of shared and intersecting pathways in both Parkinson’s disease and cancer (40). Parkinson’s disease is a protein-misfolding disease, in which misfolded α-synuclein aggregates and accumulates in Lewy bodies, leading to neurodegeneration (41). In cancer, protein misfolding and aggregation can activate oncoproteins or inactivate tumor suppressors, leading to tumorigenesis (42). As such, the autophagy-lysosome pathway can help degrade protein aggregates and organelles by autophagosome engulfment and fusion to lysosomes that contain hydrolases. Modulators of this pathway, such as 17-AAG and rapamycin, have been shown to be effective in reducing α-synuclein in cells (43, 44). Furthermore, autophagy regulation in animal models has been shown to suppress tumor formation (45). Temsirolimus, a rapamycin derivative, was approved for the treatment of renal cell carcinoma (46).

Autophagy modulators have been used in the setting of brain metastases (31). The addition of chloroquine to whole-brain radiation significantly improved median progression-free survival and decreased mortality related to brain disease progression in comparison with controls, with no increase in toxicity (47). Nevertheless, this strategy did not control extracranial disease and did not significantly increase overall survival (47). A major limitation of autophagy modulators, such as chloroquine, in the brain tumor setting is low BBB permeability (48, 49). Higher concentrations are needed to achieve the desired autophagic effects, which can become toxic (50). As such, metixene — a CNS agent that is BBB permeable and FDA approved with a low toxicity profile — can be a promising therapeutic option in clinical settings of BCBM, as it has shown efficacy against primary and metastatic breast cancer cells in vitro and in vivo and intracranially and extracranially.

Our results demonstrated that metixene activated autophagy but suppressed autophagic degradation, a process known as incomplete autophagy. Incomplete autophagy is an impaired self-eating process of intracellular macromolecules, in which generated and accumulated autophagic vesicles do not degrade, resulting in the blockage of autophagic flux (18). It has been reported that incomplete autophagy plays a crucial role in disrupting cellular homeostasis and promotes only cell death (18). Our results confirm that the elevation of LC3II upon metixene treatment with or without chloroquine is indicative of efficient induction of autophagosome formation (22, 23). Moreover, p62 accumulation proves autophagic vesicle accumulation and inhibition of autophagy completion (23). Most of the recognized compounds that affect autophagy typically act as either inducers or inhibitors of the process; few can do both functions. Drugs capable of modulating autophagy through dual mechanisms, both induction and inhibition, have emerged as a promising strategy in anticancer therapy (51–53). These compounds can elicit metabolic energy depletion, ultimately resulting in cellular stress and apoptosis (53). Furthermore, the accumulation of autophagic vesicles containing undegraded cargo, which cannot be effectively recycled, can exacerbate cellular stress (53).

NDRG1 has been reported to act as a metastasis suppressor in multiple human cancers (54–57), including breast cancer (58). Its upregulation can induce tumor differentiation and inhibit metastasis and cell proliferation (54, 59). Its potent anticancer effects have made it an important target for therapy. Iron chelators, such as Dp44mT and DpC, which exhibit selective antitumor activity were shown to upregulate NDRG1 and inhibit stress-induced autophagy in cancer cells (60, 61). In our work, we found that metixene upregulated NDRG1 expression and phosphorylation, which led to metastasis suppression through incomplete autophagy and apoptosis (Supplemental Figure 16).

The role of NDRG1 in human cancer has sparked controversy, given its dual nature. While it exhibits metastasis-suppressing properties in many cancer types, it has also been identified as a biomarker associated with metastasis, cancer recurrence, and poor prognosis in a few other cancer types (62). In the context of breast cancer, NDRG1 appears to be multifaceted, as it has been proposed to act as a promoter of tumor growth and brain metastasis in some cases of estrogen receptor–negative (ER-negative), aggressive breast cancers (63, 64). Analysis of multiple independent data sets has shown that NDRG1 levels are statistically significantly higher in tumors with aggressive molecular subtypes, such as triple-negative breast cancers (65). In our work, we show that overexpression of NDRG1 with metixene beyond the baseline elevated levels in triple-negative breast cancer cell lines and brain metastases, such as MDA-MB-231Br, induced incomplete autophagy, leading to decreased cell viability in vitro and improvement of survival in multiple preclinical models in vivo.

Further investigation and experimentation are needed to provide a comprehensive understanding of how the molecular mechanisms of metixene are interconnected in the context of cancer cell biology. Following cancer cell exposure to metixene, several coordinated processes unfold. Metixene induces substantial DNA damage, while concurrently impairing DNA repair mechanisms, as evidenced by the downregulation of DNA damage repair proteins. The surge in DNA damage may increase cellular stress and induce the expression and phosphorylation of NDRG1, a marker of the cellular stress response. Incomplete autophagy, driven by NDRG1 upregulation, further exacerbates the accumulation of damaged cellular constituents, overwhelming the cell and prompting its elimination via apoptosis. This process may further disrupt the self-renewal capabilities of cancer stem cells by accumulating damaged DNA or organelles, ultimately leading to their differentiation or death. This intricate network underscores the efficacy of metixene in dismantling cancer cells by targeting DNA repair machinery, stem-like properties, and invoking cellular stress responses, providing a comprehensive and nuanced approach to its anticancer effect.

In conclusion, our study provides preclinical, translational evidence of the effectiveness of metixene as a therapeutic agent against metastatic cancer and brain metastases, while offering insights on its mechanism of action. Our findings warrant further exploration and the potential translation of metixene use to the clinic.

## Methods

### Cell culture.

HCC1954, HCC3153, and HCC1806 cells were cultured in RPMI-1640 medium (Corning) supplemented with 10% FBS (Hyclone). MDA-MB-231, MDA-MB-231BrM2 (referred to herein as MDA-MB-231Br), BT-474, BT-474BrM3 (referred to herein as BT-474Br), and HS578T cells were cultured in DMEM medium (Corning) with 10% FBS. SUM159PT cells were maintained in F12 medium (Corning) supplemented with 5% FBS, 1 μg/mL hydrocortisone, 10 mM HEPES, and 5 μg/mL insulin. All cells were supplemented with 1% penicillin/streptomycin (Invitrogen, Thermo Fisher Scientific) and cultured at 37°C in a humidified (5%) CO_2_ incubator. MDA-MB-231BrM2 and H2030Br cell lines were provided by Joan Massagué (Memorial Sloan Kettering Cancer Center, New York, New York, USA). BT-474 and BT-474BrM3 cell lines were provided by Dihua Yu (MD Anderson Cancer Center (Houston, Texas, USA). HCC1954, HCC3153, HCC1806, MDA-MB-231, and HS578T cell lines were purchased from the American Type Culture Collection (ATCC). The SUM159PT cell line was purchased from BIOVT. The WM3734 cell line was purchased from Rockland. Cells were screened periodically for mycoplasma contamination. Metixene hydrochloride was obtained from MedChemExpress.

### CNS small-molecule inhibitor screening.

The Prestwick CNS Drug Library of 320 CNS compounds was purchased from Prestwick Chemical Libraries for screening. Cells were seeded at a density of 5,000 cells/well in clear, flat-bottomed, black-walled 96-well plates coated with laminin and poly-d-lysine. Culture medium and differentiation factors were the same as in previous assays. Each compound from the library was added at a final concentration of 25 μM per well 1 day after seeding of cells. The screens were run with concentrations ranging from 5 to 100 μM. The 5 μM concentration failed to identify a sufficient number of meaningful hits. High concentrations (50 and 100 μM), on the other hand, identified numerous hits, probably as a result of nonspecific toxicity. As such, 25 μM was chosen as a moderate concentration for screening. After 3 days of treatment, cell viability was measured using the CellTiter-Glo Luminescent Cell Viability Assay (Promega) according to the manufacturer’s instructions. Briefly, cells were incubated with an equal volume of Cell Titer Glo reagent and incubated for 10 minutes, and then luminescence was measured using Cytation 5 (Agilent Technologies) according to the manufacturer’s instructions.

### Cleaved caspase-3/-7 assay.

Cells were plated in a 96-well plate. The next day, cells were treated with metixene at different concentrations (control, 5 μM, 10 μM, and 15 μM) for 24 or 48 hours. Apoptosis was measured using the Caspase-Glo 3/7 Assay System (Promega) according to the manufacturer’s instructions. Briefly, cells were incubated with an equal volume of caspase-3/-7 Glo reagent and incubated for 10 minutes, and then luminescence was measured using Cytation 5 according to the manufacturer’s instructions.

### Caspase-8/-9 assays.

Cells were plated in a 96-well plate. The next day, cells were treated with metixene at different concentrations (control, 5 μM, 10 μM, and 15 μM) for 24 hours. Apoptosis was measured using the Caspase-Glo 8 Assay System and the Caspase-Glo 9 Assay System (Promega) according to the manufacturer’s instructions. Briefly, cells were incubated with an equal volume of Caspase-8 or Caspase-9 Glo reagent and incubated for 20 minutes, and then luminescence was measured using Cytation 5 according to the manufacturer’s instructions.

### Western blot analysis.

Western blotting was performed using standard protocols. Briefly, after treatment with metixene, chloroquine, and/or wortmannin, cells were lysed with M-PER Buffer (Thermo Fisher Scientific) containing protease and phosphatase inhibitors (Thermo Fisher Scientific). After electrophoresis, the blots were blocked for 1 hour at room temperature with 5% milk in 10 mM Tris-HCl, pH 7.5, 150 mM NaCl containing 0.1% Tween 20 (TBST) and incubated at room temperature for 1 hour with primary antibodies reactive to cleaved caspase-3 (9661, Cell Signaling Technology); β-actin (4967, Cell Signaling Technology); LC3A/B (4108, Cell Signaling Technology); p-NDRG1 (5482, Cell Signaling Technology); NDRG1 (5196, Cell Signaling Technology); SQSTM1/p62 (5114, Cell Signaling Technology); and GAPDH (2118, Cell Signaling Technology). After washing, the blots were incubated with donkey anti–rabbit IgG (H+L) cross-adsorbed secondary antibody and HRP (1:5,000, 31458, Thermo Fisher Scientific) for 1 hour at room temperature. The antigen-antibody reaction was detected using the ECL prime kit (MilliporeSigma) according to the manufacturer’s instructions. See the complete, unedited blots in the supplemental material.

### Immunofluorescence.

Cells were grown on coverslips for 48 hours until they reached 70% confluence. Then, adherent cells were washed twice with PBS and fixed with methanol for 15 minutes at –20^o^C for LC3, or in 2% paraformaldehyde (PFA) for 30 minutes in the case of cleaved caspase-3. Then, cells were permeabilized using 0.3% Triton X-100 for 10 minutes and blocked in 5% BSA for 1 hour at room temperature. Primary antibodies against cleaved caspase-3 (9661, Cell Signaling Technology) or LC3A/B (4108, Cell Signaling Technology) were incubated for 1 hour at room temperature. After 3 washes in PBS, cells were incubated with an anti–rabbit Alexa Fluor 568–conjugated secondary antibody for 1 hour. Cells were then washed in PBS thrice, and slides were mounted in DAPI mounting medium (ProLong Gold, Thermo Fisher Scientific) and imaged with a Leica DMi8 epifluorescence microscope. For in vivo cleaved caspase-3 detection, brains from mice previously injected with brain-metastatic breast cancer cells and that had received the indicated in vivo treatments were harvested, embedded in OCT, and sectioned. Sections (5 μm thick) were washed in PBS, fixed with 4% PFA for 15 minutes, and permeabilized with 0.3% Triton X-100 (MilliporeSigma) for 10 minutes. Nonspecific binding sites were blocked using 5% BSA for 1 hour and then incubated overnight with anti–human cleaved caspase-3 (9661, Cell Signaling Technology). After intermittent washes in PBS, sections were incubated with an anti–rabbit secondary antibody coupled to Alexa Fluor 555 for 1 hour at room temperature. After washing in PBS, the slides were mounted in DAPI mounting medium (ProLong Gold) and imaged with a Leica DMi8 epifluorescence microscope. The quantification of fluorescent areas was conducted using ImageJ software (NIH) (66).

### mCherry-GFP-LC3.

FUW mCherry-GFP-LC3 was a gift from Anne Brunet (Addgene plasmid 110060; http://n2t.net/addgene:110060; RRID: Addgene_110060) (67). Cells at 70% confluence were transiently transfected with the plasmid using Lipofectamine 2000 (Invitrogen, Thermo Fisher Scientific). Following 24 hours of transfection, the media were changed, and cells were sorted to isolate those that expressed the mCherry-GFP configuration. Confocal fluorescence images were collected with a Nikon AXR point scanning confocal fluorescence microscope (Nikon Instruments) using a Nikon Plan Apo λD ×60/NA 1.42 Type F oil immersion objective and GaAsP point detectors. All images were collected with a pinhole size of 32.6 μm corresponding to 1.0 AU at 561 nm excitation and a 4.0 μs dwell time for all channels. DAPI was imaged with 405 nm excitation and a 430–475 nm emission range, a detector gain factor of 34.0, and nominal laser power of 4.0%. GFP was imaged with 488 nm excitation and 499–530 nm emission, a detector gain factor of 34.0, and nominal laser power of 5.0%. mCherry was imaged with 561 nm excitation and 600–635 nm emission, a detector gain factor of 30.0, and nominal laser power of 4.0%. Volume *Z*-stack series were collected over a total volume of 9.4 μm in depth, corresponding to 48 total frames of 512 × 512 pixels in each frame with a voxel size of 0.11 × 0.11 × 0.2 μm. All images were collected with a pinhole size of 32.6 μm corresponding to 1.0 AU at 561 nm excitation and a 4.0 μs dwell time for all channels, using NIS Elements Software, version 5.41.

### Electron microscopy.

Cell culture samples on Thermanox plastic in a 24-well plate were fixed in 0.1 M sodium cacodylate buffer, pH 7.35, containing 2% PFA and 2.5% glutaraldehyde and postfixed with 2% osmium tetroxide in unbuffered aqueous solution; rinsed with distilled water; en-bloc stained with 3% uranyl acetate; rinsed with distilled water; dehydrated in ascending grades of ethanol; transitioned with a 1:1 mixture of ethanol and resin; and embedded in a resin mixture of the Embed 812 kit and cured in a 60°C oven. Samples were sectioned on a Leica Ultracut UC6 ultramicrotome. Sections (70 nm thick) were collected on 200 mesh copper grids and poststained with 3% uranyl acetate and Reynolds lead citrate. Photos were obtained with a FEI Tecnai Spirit G2 transmission electron microscope at 120 kV.

### IHC.

Murine brain tissues were embedded in paraffin and cut into 4 μm thick sections on positively charged slides. Dewaxed sections were routinely stained with H&E dyes for histological evaluation and were also processed for chromogenic immunohistochemistry for the following primary rabbit antibodies: LC3B (catalog ab48394, Abcam; dilution: 1:600); cleaved caspase-3 (catalog ab4051, Abcam; dilution: 1:200); and p-NDRG1 (catalog 5482, Cell Signaling Technology; dilution: 1:500). An anti–rabbit antibody–HRP polymer conjugate (MACH2, Biocare) was used in conjunction with the chromogenic substrate DAB to visualize the primary antibody sites. IHC slides were counterstained with hematoxylin. The quantification of stained areas was conducted using ImageJ software (66).

### qRT-PCR.

RNA was extracted using RNAeasy kit from QIAGEN according to the manufacturer’s protocol. cDNA was prepared from 1 μg RNA using iScript cDNA synthesis kit (Bio-Rad) according to the manufacturer’s protocol. The cDNA was then subjected to quantitative real-time PCR (qRT-PCR) in the Bio-Rad CFX Connect system. The primer sequences used can be found in the Supplemental Methods.

### Generation of NDRG1-KO cells.

Single-gene–KO clones were generated in lentiCRISPRv2 (1-vector system). The vector backbone was purchased from Addgene (lentiCRISPR v2 was a gift from Feng Zhang; Addgene plasmid 52961; http://n2t.net/addgene: 52961; RRID: Addgene_52961) (68). The protocol for guide cloning and generation of the virus was as described previously (68). The guide sequence for human NDRG1 KO was CCTGCAAGAGTTTGATGTCC, and the nontargeting control (VC) sequence was AATATTTGGCTCGGCTGCGC. The NDRG1-KO and control clones were selected using puromycin from MilliporeSigma (1 μg/mL) in the MDA-MB-231Br cell line. NDRG1 KO was confirmed by Western blotting (NDRG1 antibody, 5196, Cell Signaling Technology).

### Animal experiments.

Six- to 8-week-old athymic, immunodeficient^(nu/nu)^ female mice were obtained from Charles River Laboratories and maintained in a specific pathogen–free facility. Mice were anesthetized with an intraperitoneal injection of 100 μL stock solution containing ketamine HCl (25 mg/mL) and xylazine (2.5 mg/mL).

For mammary gland injections, 1 × 10^6^ HCC1954 cells were diluted 1:1 with Matrigel Matrix (BD Biosciences) for a final volume of 40 μL and injected into the inguinal mammary fat pad of nude female mice. Two weeks after tumor cell injection, mice were randomly divided into the following 3 treatment groups: vehicle (25% captisol, i.p.), metixene (0.1 mg/kg, i.p.), and metixene (1.0 mg/kg, i.p.). Higher doses of 5 mg/kg and 10 mg/kg were toxic to the mice. Tumor growth was monitored once a week by caliper measurement.

For the stereotactic intracranial injection, the surgical site was prepared with 70% ethyl alcohol. A midline incision was made, and a 1 mm diameter parietal burr hole, centered 2 mm posterior to the coronal suture and 2 mm lateral to the sagittal suture, was drilled. Mice were placed in a stereotactic frame, and 5 × 10^5^ BT-474Br cells previously resuspended in 5 μL sterile PBS were intracranially injected with a 26 gauge needle at a depth of 3 mm. The needle was removed, and the skin was sutured with a 4-0 absorbable, synthetic braided suture. After 10 days, the mice were randomly divided into the following 2 treatment groups: vehicle (25% captisol, i.p.) and metixene (1.0 mg/kg, i.p.). Mice were treated 3 times per week and monitored for 3 months or upon meeting the established IACUC criteria for euthanasia (69). Brain tissues were embedded in OCT freezing reagent, and sections with a thickness of 4 μm were cut, air-dried, and stained with H&E.

For the intracardiac injection of cancer cells, the surgical site was prepared with 70% ethyl alcohol to ensure aseptic conditions. Subsequently, the mice were placed in the supine position, and a suspension containing 2.5 × 10^5^ MDA-MB-231 cells, resuspended in 100 μL sterile PBS, was loaded into a 26 gauge needle for precise intracardiac delivery into the left ventricle of the heart. To verify the accurate placement of the needle within the left ventricle, 10–20 μL fluid was aspirated, with confirmation indicated by the presence of bright red (arterial) blood. Once proper placement was confirmed, the cancer cell suspension was administered gradually over a 30-second interval. After a 7-day incubation period, the mice were randomly allocated to 1 of 2 treatment cohorts: (a) the vehicle treatment group, receiving 25% captisol via intraperitoneal injection or (b) the metixene treatment group, subjected to intraperitoneal injections of metixene at a dose of 1.0 mg/kg. Treatment was administered thrice weekly, and continuous monitoring of the mice ensued until they met the predetermined euthanasia criteria, as established by the IACUC (69). Following euthanasia, organ tissues were promptly embedded in 4% PFA reagent, followed by the preparation of 4 μm thick sections. These sections were subsequently air-dried and subjected to H&E staining for histological examination.

### Intracarotid artery injections.

The brain-metastatic MDA-MB-231Br cells were harvested and passed through a 70 μM strainer to obtain single cells. The cells were then centrifuged at 350*g* for 5 minutes and washed with cold PBS. The cells were counted and aliquoted, with 1 million cells in 200 μL cold PBS. Six- to 8-week-old nude mice were anesthetized by intraperitoneal injection of ketamine/xylazine cocktail and positioned in dorsal recumbency. Lubricant was applied to the eyes to prevent corneal ulcer formation. The surgical field at the ventral side of the neck was draped and sterilized by applying povidone-iodine followed by application of a 70% alcohol solution. A 1 cm long skin incision was created using a surgical blade. The underlying subcutaneous tissue was gently dissected, and the sternohyoid muscle was bluntly separated from the sternomastoid muscle to expose the left common carotid artery. Then, the digastric muscle was bluntly separated to expose the external and internal branches of the common carotid artery. Furthermore, the vagus nerve was gently separated from the carotid artery using surgical forceps under a dissection microscope. A tight surgical knot was placed at the caudal end of the common carotid artery. A cotton ball dipped in sterile PBS was placed below the carotid artery to assist in injection and to maintain moisture over the artery. A second surgical knot was placed over the rostral end of the common carotid artery. Before injection, a hemoclip was placed over the external branch of the common carotid artery. A 31 gauge BD insulin syringe was inserted into the lumen of the artery followed by infusion of 100 μL cells. Once the infusion of cells was completed, the common carotid artery was permanently ligated at its rostral end to prevent the leakage of cells. Previously bluntly separated muscles were moved to the original sites, and subcutaneous tissue and skin were closed with 6-0 size nylon surgical sutures in a simple interrupted fashion.

### Bioluminescence imaging.

The brain-metastatic capabilities of BT-474BrM3-mCherry-Luc cells were monitored biweekly. Mice were administered luciferin (15 mg/mL, in sterile PBS) (GoldBio) through intraperitoneal injection. The mice were sedated with isoflurane anesthesia and placed in the bioluminescence camera box of an IVIS spectrum imager for approximately 10 minutes, and bioluminescence was measured.

### Pharmacokinetics and bioavailability.

Sample preparation entailed a meticulous acetonitrile-based protein crash procedure with incorporation of the internal standard (Methadone) for precise quantification. Quantification was facilitated through a matrix-matched curve spanning a dynamic range from 1 to 1,000 ng/mL in both plasma and brain homogenate samples. To ensure data accuracy, samples were analyzed in triplicate, and the reported results represent the average of these measurements.

Mass spectrometric data acquisition was conducted using the Agilent 6475 LC/TQ instrument, utilizing MassHunter version 12 acquisition software, and subsequent data processing was performed with the MassHunter version 12 Quantitative Analysis Tool. The mass spectrometer was seamlessly integrated with the Agilent 1290 Infinity II UPLC system. During analysis, the samples were subjected to a gradient method, with a precisely controlled injection volume of 1 μL. The positive detection mode was used, with methadone serving as the internal standard for calibration and quantification purposes. Chromatographic separation was meticulously executed utilizing the InfinityLab Poroshell 120 EC-C18 column, measuring 2.1 × 100 mm and featuring a 1.9 μm particle size. The mobile phase composition encompassed 0.1% formic acid in water (A) and 0.1% formic acid in acetonitrile (B). The gradient method was characterized by a flow rate of 0.45 mL/min, which facilitated a transition from an initial 30% B composition to a final 100% B composition over a span of 6 minutes.

### RPPA.

An RPPA was performed at the MD Anderson Cancer Center (MDACC) Cancer Center Support Grant (CCSG) core as described at http://www.mdanderson.org/education-and-research/resources-for-professionals/scientific-resources/core-facilities-and-services/functional-proteomics-rppa-core/index.html

Morpheus (https://software.broadinstitute.org/morpheus), a versatile matrix visualization and analysis software, was used for to generate heatmaps.

### Statistics.

Statistical analyses were performed using GraphPad Prism version 8 for Windows (GraphPad Software), SAS 9.4 (SAS Institute), and R version 3.5.2 (R Foundation for Statistical Computing). For continuous variables, data are reported as the mean ± SEM. For categorical variables, data are reported as numbers (percentages). For continuous variables, depending on whether the assumption of normality and the assumption of equal variance were met, a 2-tailed, unpaired Student’s *t* test (when both assumptions were met) or an unpaired Student’s *t* test with Welch’s correction (when only the assumption of equal variance was not met) was used for 2 unpaired groups. One-way or 2-way ANOVA with Tukey’s or Dunnett’s multiple-comparison test was performed for multiple-group comparisons. For data with 2 independent variables, for example, treatment and time, a linear mixed model was fitted, and the differences among groups were evaluated using the least-squares means method and adjusted by Šidák’s or Dunnett’s method. Kaplan-Meier survival curves were plotted and compared using the log-rank test for proportional hazard rates or Renyi statistics for crossing hazard rates. A *P* value of less than 0.05 was considered significant.

### Study approval.

All animal studies were conducted according to the NIH guidelines on the care and use of laboratory animals for research purposes and were approved by the IACUC Office of Northwestern University (protocol 2559).

### Data availability.

All data generated or analyzed during this study were included in this article (and its supplemental materials). Raw data for the manuscript are also available in the Supplemental Supporting Data Values file.

## Author contributions

JF and MSL conceptualized the study, provided resources, supervised the study, validated results, performed visualization, designed study methodology, and were responsible for administration of the project. JF curated the data and performed formal analysis. MSL acquired funding for the study. JF wrote the original draft of the manuscript. JF, EP, DK, CD, AC, JTD, RY, MHS, PZ, AR, VAA, IU, AUA, JM, IVB, CDJ, AMS, ABH, and MSL performed experiments and revised the final draft of the manuscript.

## Supplementary Material

Supplemental data

Supporting data values

## Figures and Tables

**Table 2:**
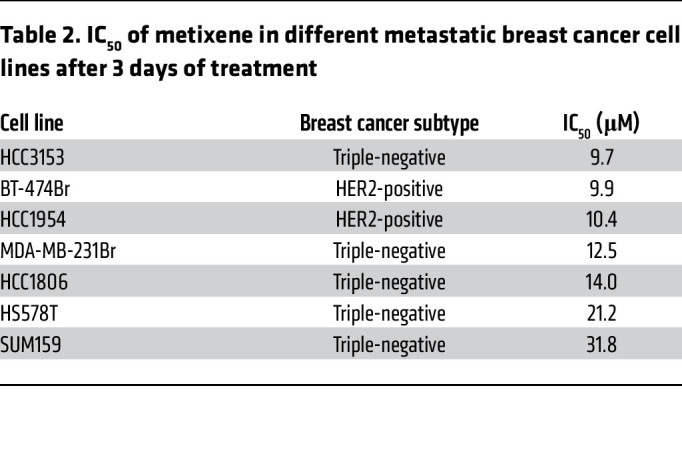
IC_50_ of metixene in different metastatic breast cancer cell lines after 3 days of treatment

**Table 1:**
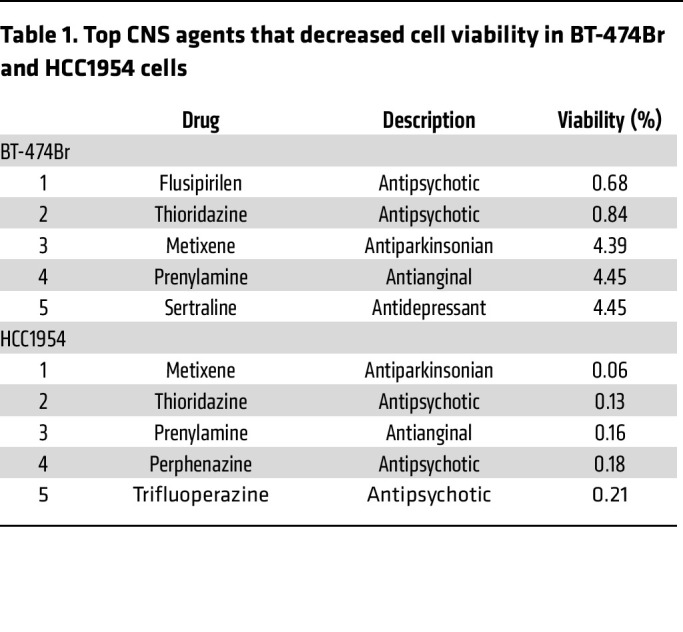
Top CNS agents that decreased cell viability in BT-474Br and HCC1954 cells

## Version 1 10/17/2023

In-Press Preview

## Version 2 12/15/2023

Electronic publication
