# Supplementary material for: Metixene is an incomplete autophagy inducer in preclinical models of metastatic cancer and brain metastases
Source: J Clin Invest. 2023 Dec 15;133(24):e161142. doi: 10.1172/JCI161142 (PMC10721147; doi:10.1172/JCI161142)

Supplemental Material

Metixene is an incomplete autophagy inducer in preclinical models of metastatic cancer and brain metastases

Fares et al.

Supplementary Methods **2**
Supplementary Tables **3**
Supplementary Table 1 3
Supplementary Table 2 8
Supplementary Figures **11**
Supplementary Figure 1 11
Supplementary Figure 2 12
Supplementary Figure 3 13
Supplementary Figure 4 14
Supplementary Figure 5 15
Supplementary Figure 6 16
Supplementary Figure 7 17
Supplementary Figure 8 18
Supplementary Figure 9 19
Supplementary Figure 10 20
Supplementary Figure 11 21
Supplementary Figure 12 22
Supplementary Figure 13 23
Supplementary Figure 14 24
Supplementary Figure 15 25
Supplementary Figure 16 26

Supplementary Methods

The primer sequences are as follows:

Primer	Sequence
CHRM1-F	AGAAAGGGCGTGATCGAGC
CHRM1-R	CTTGACCAGCGAGAAGGTCTT
CHRM2-F	TGGTTTCCATTAAAGTCAACCGC
CHRM2-R	ACACCTATGATAAGGTCAGCACA
CHRM3-F	CACGTACCGAGCCAAACGAA
CHRM3-R	AGGACAAAGGAGATGACCCAA
CHRM4-F	AGGACACTTCCAATGAGTCCA
CHRM4-R	TGTCTGCTTCGTCACAATCTG
CHRM5-F	AGTCTGGCTTGTGACCTTTGG
CHRM5-R	TGTCAAGGGTCTTGTGATGGA
HRH3-F	CCCATACACGCTGCTGATGAT
HRH3-R	GGAGGTTTCGTACCAGTAGTCA

Supplementary Table 1

Prominent signaling pathways identified by RPPA after 12 hours of treatment of BT-474Br cells with metixene hydrochloride (10µM)

(Blue text ➔ Gene overexpression)

1. MAPK Signaling
2. PI3K/Akt Signaling
3. Apoptosis Signaling
4. Autophagy Signaling
5. MTOR Signaling
6. DNA Damage and Repair
7. P53 Pathway
8. Cell Cycle Control
9. Energy Metabolism
10. Ubiquitination and Proteasome Signaling
11. Cellular Response to Stress

**MAPK Signaling (12 hours)**				
**Number**	**Gene**	**Phosphorylation**	**P value**	**Fold**
1	RPS6KB1	p70-S6K_pT389	9.38272E-06	2.507056491
2	DUSP4	-	0.000908562	2.274395435
3	F2RL1	-	8.21967E-05	2.223662267
4	CREB1	CREB_pS133	4.91133E-06	1.805124858
5	GSK3A	GSK-3a-b_pS21_S9	3.28172E-05	1.726581413
6	CDKN1A	-	0.000188502	1.626810371
7	RB1	Rb_pS807_S811	0.000228951	1.536820339
8	PLCG1	-	6.99047E-05	1.37381962
9	KDR	VEGFR-2_pY1175	0.000363087	1.367836777
10	ACACA	-	4.10702E-05	1.33534115
11	JUN	c-Jun_pS73	6.08211E-05	1.327826432
12	EPHA2	EphA2_pY588	8.60967E-05	1.316669568
13	AKT1	Akt_pS473	0.000178338	1.30173633
14	MTOR	mTOR_pS2448	2.34749E-05	1.299443666
15	IRS2	-	0.000408732	1.282253448
16	HSPB1	HSP27_pS82	0.000235328	1.27832769
17	DUSP6	-	0.00023118	1.24117736
18	RPS6KB1	-	0.000424264	1.220282382
19	FRS2	FRS2-alpha_pY196	0.000378889	1.177194692
20	DDR1	-	0.000315905	1.172097761
21	RPS6KA1	p90RSK_pT573	7.6095E-05	0.849870689
22	ERBB3	-	0.000180729	0.779807018
23	MYT1	-	0.000235871	0.744359953
24	ESR1	-	0.000715034	0.695598229

PI3K/Akt Signaling (12 hours)				
**Number**	**Gene**	**Phosphorylation**	**P value**	**Fold**
1	RPS6KB1	p70-S6K_pT389	9.38272E-06	2.507056491
2	CREB1	CREB_pS133	4.91133E-06	1.805124858
3	GSK3A	GSK-3a-b_pS21_S9	3.28172E-05	1.726581413
4	CDKN1A	-	0.000188502	1.626810371
5	RICTOR	Rictor_pT1135	6.92021E-05	1.462210688
6	WEE1	-	3.81148E-05	1.392015151
7	YAP1	YAP_pS127	0.000311251	1.376021777
8	KDR	VEGFR-2_pY1175	0.000363087	1.367836777
9	JUN	c-Jun_pS73	6.08211E-05	1.327826432
10	EPHA2	EphA2_pY588	8.60967E-05	1.316669568
11	AKT1	Akt_pS473	0.000178338	1.30173633
12	MTOR	mTOR_pS2448	2.34749E-05	1.299443666
13	IRS2	-	0.000408732	1.282253448
14	RPS6KB1	-	0.000424264	1.220282382
15	FRS2	FRS2-alpha_pY196	0.000378889	1.177194692
16	ERBB3	-	0.000180729	0.779807018
17	MYT1	-	0.000235871	0.744359953

Apoptosis Signaling (12 hours)				
**Number**	**Gene**	**Phosphorylation**	**P value**	**Fold**
1	RPS6KB1	p70-S6K_pT389	9.38272E-06	2.507056491
2	TNFRSF12A	-	1.08011E-05	1.564722447
3	KDR	VEGFR-2_pY1175	0.000363087	1.367836777
4	JUN	c-Jun_pS73	6.08211E-05	1.327826432
5	AKT1	Akt_pS473	0.000178338	1.30173633
6	HSPB1	HSP27_pS82	0.000235328	1.27832769
7	CHEK2	-	0.000486859	1.228152442
8	RPS6KB1	-	0.000424264	1.220282382
9	ABL1	-	0.000480916	0.913306016
10	ATR	ATR_pS428-R-C	0.000636469	0.911323484
11	RPS6KA1	p90RSK_pT573	7.6095E-05	0.849870689
12	ERBB3	-	0.000180729	0.779807018
13	MLKL	-	0.000261248	0.766906745

Autophagy Signaling (12 hours)				
**Number**	**Gene**	**Phosphorylation**	**P value**	**Fold**
1	RPS6KB1	p70-S6K_pT389	9.38272E-06	2.507056491
2	MAP1LC3B	-	7.11905E-05	1.595174595
3	TNFRSF12A	-	1.08011E-05	1.564722447
4	EPHA2	EphA2_pY588	8.60967E-05	1.316669568
5	MTOR	mTOR_pS2448	2.34749E-05	1.299443666
6	IRS2	-	0.000408732	1.282253448
7	RPS6KB1	-	0.000424264	1.220282382
8	ABL1	-	0.000480916	0.913306016
9	MLKL	-	0.000261248	0.766906745
10	MFN1	-	0.000106745	0.710899333

MTOR Signaling (12 hours)				
**Number**	**Gene**	**Phosphorylation**	**P value**	**Fold**
1	RPS6KB1	p70-S6K_pT389	9.38272E-06	2.507056491
2	GSK3A	GSK-3a-b_pS21_S9	3.28172E-05	1.726581413
3	RICTOR	Rictor_pT1135	6.92021E-05	1.462210688
4	KDR	VEGFR-2_pY1175	0.000363087	1.367836777
5	AKT1	Akt_pS473	0.000178338	1.30173633
6	MTOR	mTOR_pS2448	2.34749E-05	1.299443666
7	RPS6KB1	-	0.000424264	1.220282382
8	LRP6	LRP6_pS1490	0.000710855	1.166900014
9	RPS6KA1	p90RSK_pT573	7.6095E-05	0.849870689
10	ERBB3	-	0.000180729	0.779807018

DNA Damage and Repair (12 hours)				
**Number**	**Gene**	**Phosphorylation**	**P value**	**Fold**
1	RAD17	Rad17_pS645	0.000190643	1.448604445
2	MSH6	-	4.56552E-05	1.263460077
3	CHEK2	-	0.000486859	1.228152442
4	CHD1L	-	6.41481E-06	1.132609427
5	ABL1	-	0.000480916	0.913306016
6	BAP1	-	0.000301915	0.89302666
7	TP53BP1	-	0.000216171	0.868036768
8	MLKL	-	0.000261248	0.766906745

P53 Pathway (12 hours)				
Number	Gene	Phosphorylation	P value	Fold
1	CDKN1A	-	0.000188502	1.626810371
2	RB1	Rb_pS807_S811	0.000228951	1.536820339
3	HSPB1	HSP27_pS82	0.000235328	1.27832769
4	RRM2	-	1.77271E-06	1.248385206
5	CHEK2	-	0.000486859	1.228152442
6	DDR1	-	0.000315905	1.172097761
7	ATR	ATR_pS-428-R-C	0.000636469	0.911323484
8	TP53BP1	-	0.000216171	0.868036768

Cell Cycle Control (12 hours)				
Number	Gene	Phosphorylation	P value	Fold
1	RRM2	-	1.77271E-06	1.248385206
2	CHEK2	-	0.000486859	1.228152442
3	ABL1	-	0.000480916	0.913306016
4	ATR	ATR_pS428-R-C	0.000636469	0.911323484
5	TP53BP1	-	0.000216171	0.868036768
6	MYT1	-	0.000235871	0.744359953

Energy Metabolism (12 hours)				
Number	Gene	Phosphorylation	P value	Fold
1	ACSS1	-	0.000573658	2.082199342
2	ACACA	-	4.10702E-05	1.33534115
3	GLUD1	-	0.000397735	0.82213739
4	SDHA	-	0.000973656	0.798624779
5	MFN1	-	0.000106745	0.710899333
6	ATP5PD	-	0.000230614	0.688437367

Ubiquitination and Proteasome Signaling (12 hours)				
Number	Gene	Phosphorylation	P value	Fold
1	RPS6KB1	p70-S6K_pT389	9.38272E-06	2.507056491
2	TNFRSF12A	-	1.08011E-05	1.564722447
3	RPS6KB1	-	0.000424264	1.220282382
4	SMAD1	-	0.000297995	1.091292641
5	BAP1	-	0.000301915	0.89302666
6	GATA3	-	0.000111136	0.614848105

Cellular Response to Stress (12 hours)				
**Number**	**Gene**	**Phosphorylation**	**P value**	**Fold**
1	RB1	Rb_pS807_S811	0.000228951	1.536820339
2	JUN	c-Jun_pS73	6.08211E-05	1.327826432
3	ATR	ATR_pS428-R-C	0.000636469	0.911323484
4	RPS6KA1	p90RSK_pT573	7.6095E-05	0.849870689

Supplementary Table 2

Prominent signaling pathways identified by RPPA after 24 hours of treatment of BT-474Br cells with metixene hydrochloride (10µM)

(Blue text ➔ Gene overexpression)

1. MAPK Signaling
2. PI3K/Akt Signaling
3. Apoptosis Signaling
4. Autophagy Signaling
5. MTOR Signaling
6. P53 Pathway
7. Cell Cycle Pathway
8. Chromatin Regulation
9. DNA Damage and Repair
10. Energy Metabolism

**MAPK Signaling (24 hours)**				
**Number**	**Gene**	**Phosphorylation**	**P value**	**Fold**
1	F2RL1	-	6.93E-06	2.954065229
2	MAPK1	MAPK_pT202_Y204	0.000147241	1.796775325
3	EPHA2	EphA2_pY588	0.00018709	1.537269995
4	DUSP4	-	0.000765647	1.499201084
5	KDR	VEGFR-2_pY1175	0.000118661	1.48369771
6	FRS2	FRS2-alpha_pY196	6.85718E-05	1.351922377
7	SRC	Src_pY416	0.000189853	1.344911062
8	MTOR	mTOR_pS2448	0.00059543	1.236339794
9	AKT1	Akt_pS473	0.000994206	1.234031345
10	SRC	Src_pY527	0.00050646	1.196424147
11	EEF2K	-	0.000381612	0.865541785
12	PLCG1	PLCG1_pS1248	0.000289602	0.811919733
13	MYT1	-	0.000379911	0.755027877
14	CCNB1	-	0.000714403	0.727894288
15	ESR1	-	0.000186593	0.675563142

PI3K/Akt Signaling (24 hours)				
**Number**	**Gene**	**Phosphorylation**	**P value**	**Fold**
1	TNFRSF12A	-	0.000330649	2.051109301
2	EPHA2	EphA2_pY588	0.00018709	1.537269995
3	KDR	VEGFR-2_pY1175	0.000118661	1.48369771
4	NDRG1	NDRG1_pT346	0.000722298	1.475729182
5	COL6A1	-	0.00060684	1.248962114
6	MTOR	mTOR_pS2448	0.00059543	1.236339794
7	AKT1	Akt_pS473	0.000994206	1.234031345
8	EEF2K	-	0.000381612	0.865541785
9	MYT1	-	0.000379911	0.755027877

Apoptosis Signaling (24 hours)				
**Number**	**Gene**	**Phosphorylation**	**P value**	**Fold**
1	TNFRSF12A	-	0.000330649	2.051109301
2	MAPK1	MAPK_pT202_Y204	0.000147241	1.796775325
3	KDR	VEGFR-2_pY1175	0.000118661	1.48369771
4	NDRG1	NDRG1_pT346	0.000722298	1.475729182
5	SRC	Src_pY416	0.000189853	1.344911062
6	AKT1	Akt_pS473	0.000994206	1.234031345
7	SRC	Src_pY527	0.00050646	1.196424147

Autophagy Signaling (24 hours)				
**Number**	**Gene**	**Phosphorylation**	**P value**	**Fold**
1	TNFRSF12A	-	0.000330649	2.051109301
2	EPHA2	EphA2_pY588	0.00018709	1.537269995
3	NDRG1	NDRG1_pT346	0.000722298	1.475729182
4	MTOR	mTOR_pS2448	0.00059543	1.236339794

MTOR Signaling (24 hours)				
**Number**	**Gene**	**Phosphorylation**	**P value**	**Fold**
1	KDR	VEGFR-2_pY1175	0.000118661	1.48369771
2	MTOR	mTOR_pS2448	0.00059543	1.236339794
3	AKT1	Akt_pS473	0.000994206	1.234031345
4	EEF2K	-	0.000381612	0.865541785

P53 Pathway (24 hours)				
**Number**	**Gene**	**Phosphorylation**	**P value**	**Fold**
1	EPHA2	EphA2_pY588	0.00018709	1.537269995
2	NDRG1	NDRG1_pT346	0.000722298	1.475729182
3	DDR1	-	0.000557649	1.448229987
4	CCNB1	-	0.000714403	0.727894288

Cell Cycle Pathway (24 hours)				
**Number**	**Gene**	**Phosphorylation**	**P value**	**Fold**
1	H2BU1	-	0.000722895	1.301671676
2	PLK1	-	0.000625057	0.829341124
3	MYT1	-	0.000379911	0.755027877
4	CCNB1	-	0.000714403	0.727894288

Chromatin Regulation (24 hours)				
**Number**	**Gene**	**Phosphorylation**	**P value**	**Fold**
1	H2BU1	-	0.000722895	1.301671676
2	ATRX	-	0.000439278	0.799434072
3	DNMT1	-	1.12095E-05	0.785381496

DNA Damage and Repair (24 hours)				
**Number**	**Gene**	**Phosphorylation**	**P value**	**Fold**
1	PLK1	-	0.000625057	0.829341124
2	CCNB1	-	0.000714403	0.727894288

Energy Metabolism (24 hours)				
**Number**	**Gene**	**Phosphorylation**	**P value**	**Fold**
1	LCN2	-	3.57944E-05	1.144987193
2	DNMT1	-	1.12095E-05	0.785381496

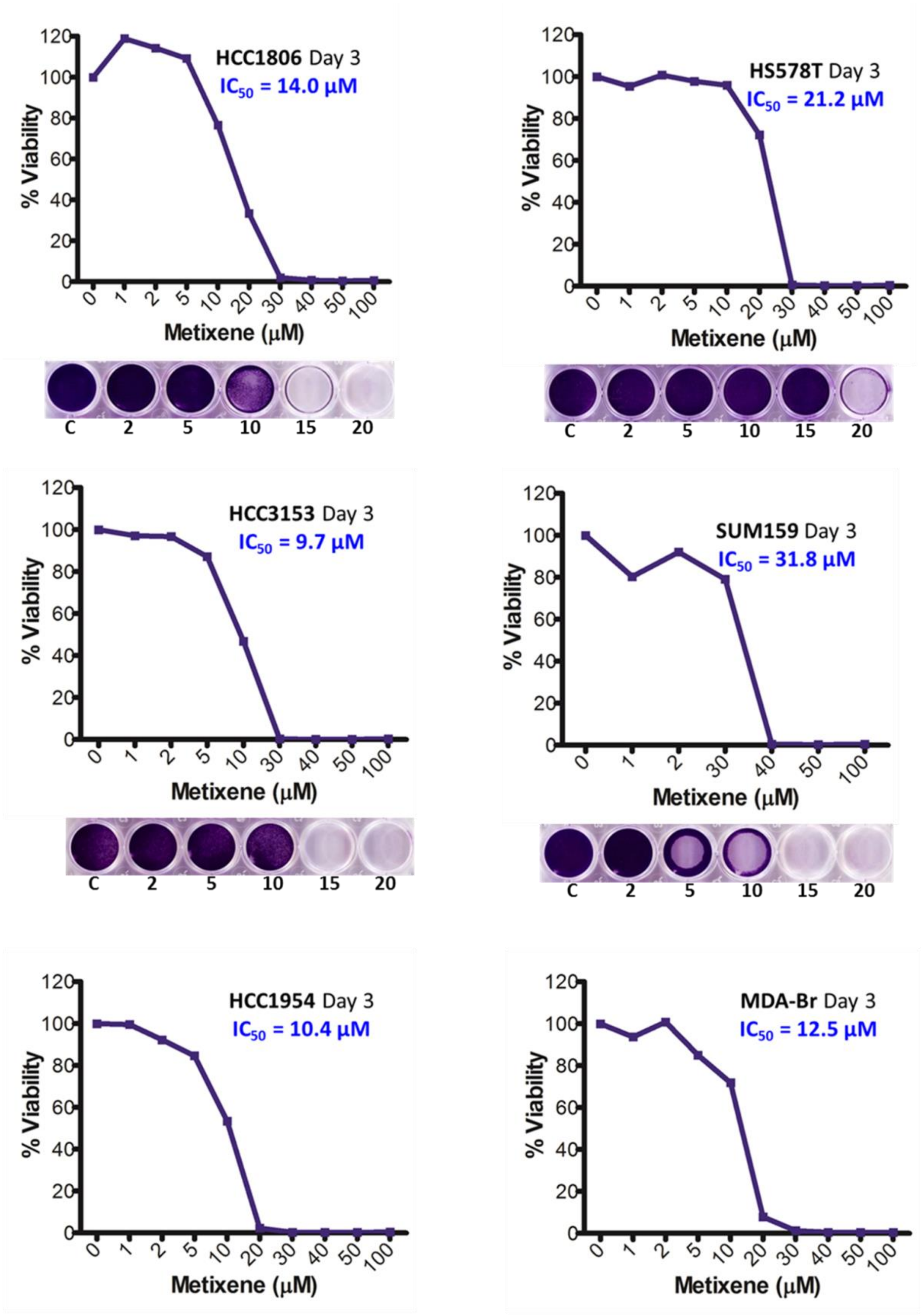

Supplementary Figure 1: IC_{50} of different metastatic breast cancer cell lines after 3 days of metixene treatment.

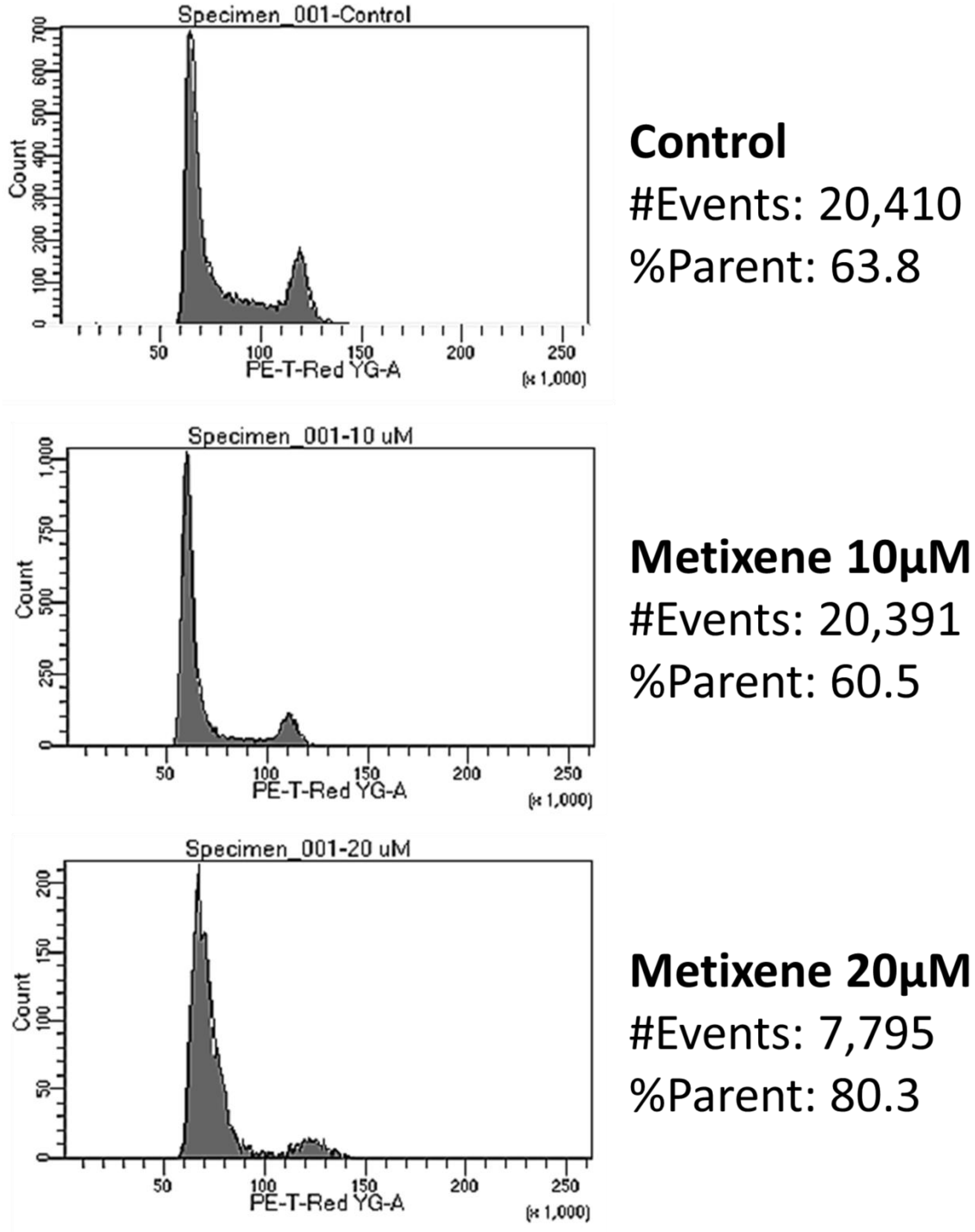

Supplementary Figure 2: Cell cycle analysis of BT-474Br cells by flow cytometry after treatment with metixene at different concentrations.

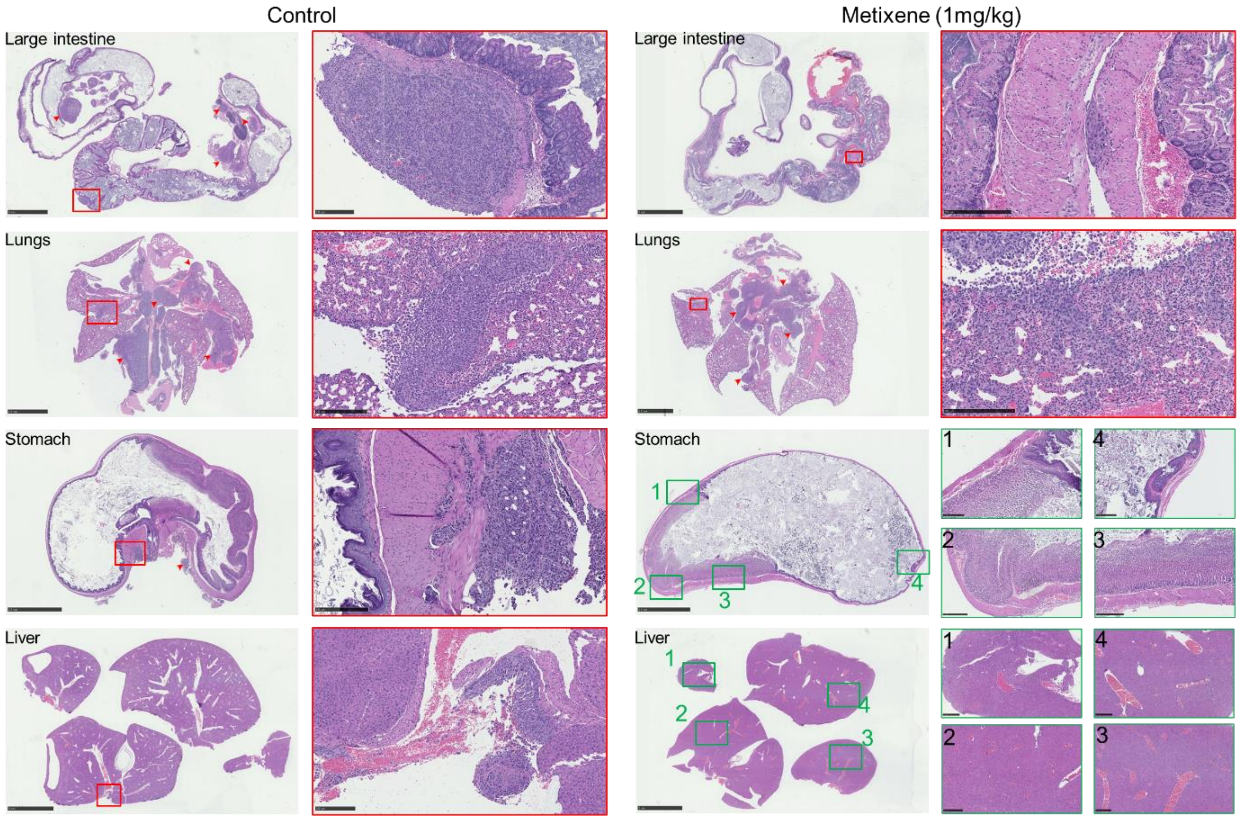

Supplementary Figure 3: Histological sections and H&E staining of visceral organs in controls (25% captisol) vs. metixene-treated mice (1 mg/kg, 3 days per week), after intracardiac injection of MDA-MB-231 cells.

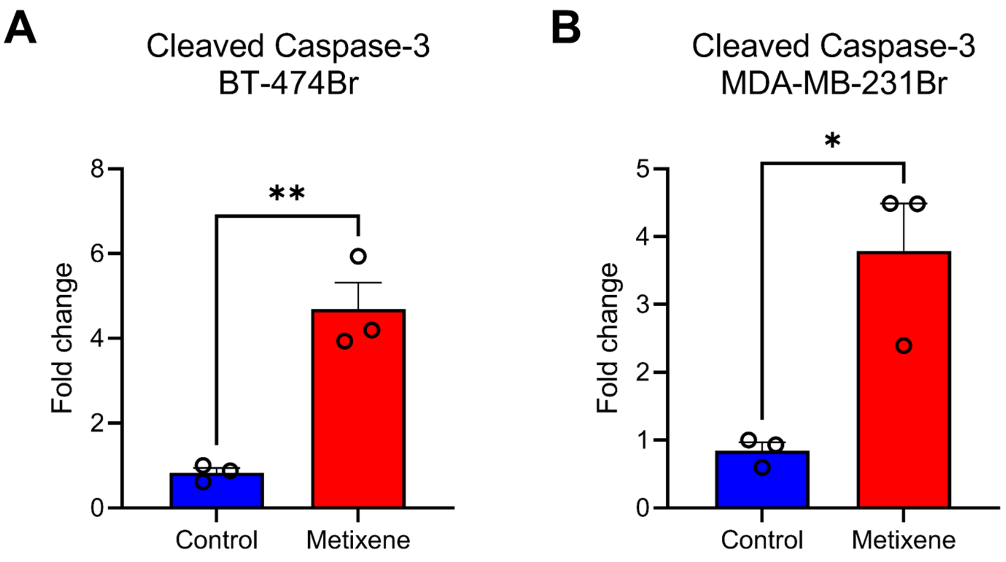

Supplementary Figure 4: Quantification of the area of cleaved caspase-3 IHC staining in the metastatic brain tumors of metixene-treated mice vs. controls after (**A**) intracranial injection of HER2-positive BT-474Br and (**B**) intracarotid injection of triple negative MDA-MB-231Br *in vivo. The results are representative of three independent experiments, and bar graphs represent means ± SEM. Statistical analysis was done using the Student's t test. ****p<0.0001; ***p<0.001; **p<0.01; *p<0.05*

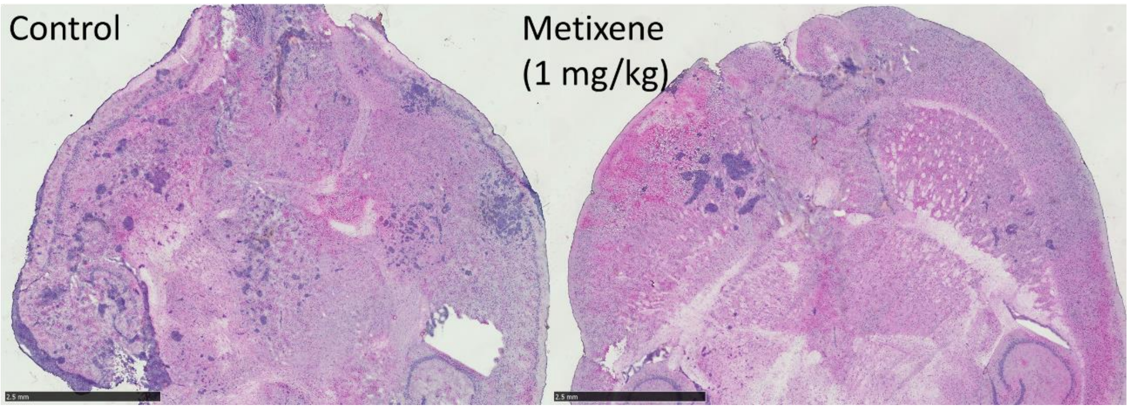

Supplementary Figure 5: H&E staining of coronal brain sections (cut in midline) of control and metixene-treated mice in a preclinical model of multiple brain metastases using MDA-MB-231Br. *Bar 2.5mm*.

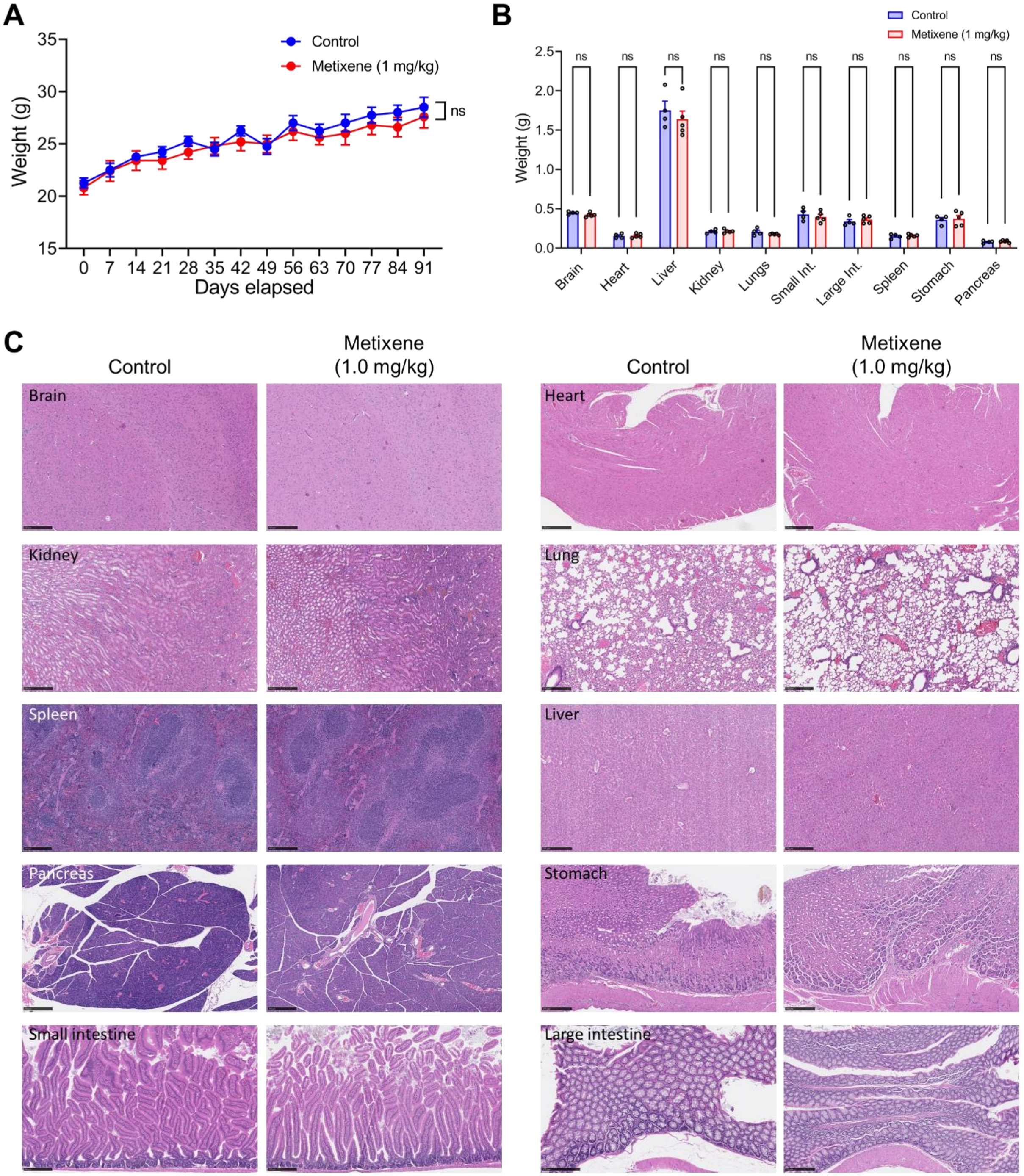

Supplementary Figure 6: Toxicity studies conducted with two groups of nontumor bearing mice: control (n=4) and metixene treated (n=4, treated at 1 mg/kg, 3 days per week). **A**, Overall body weight of control vs. metixene treated mice across time. **B**, Organ weight of control vs. metixene treated mice after 91 days. **C**, H&E staining of different organs in controls vs. metixene treated mice after 91 days. *Statistical analysis was performed using two-way ANOVA with post hoc Sidak's test. ****p<0.0001; ***p<0.001; **p<0.01; *p<0.05*

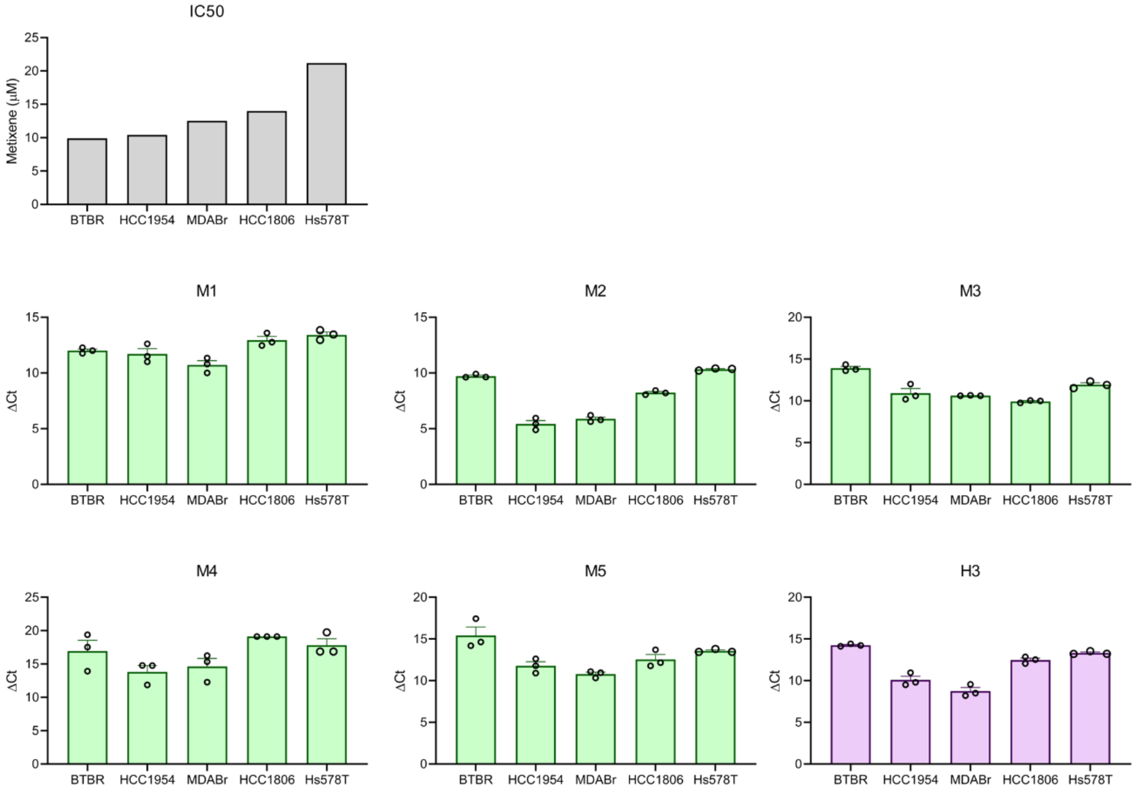

Supplementary Figure 7: IC_{50} data of several breast cancer cell lines data (grey) did not correlate with the quantitation of muscarinic receptors (green) or histaminic receptors (purple) that are expressed in the brain.

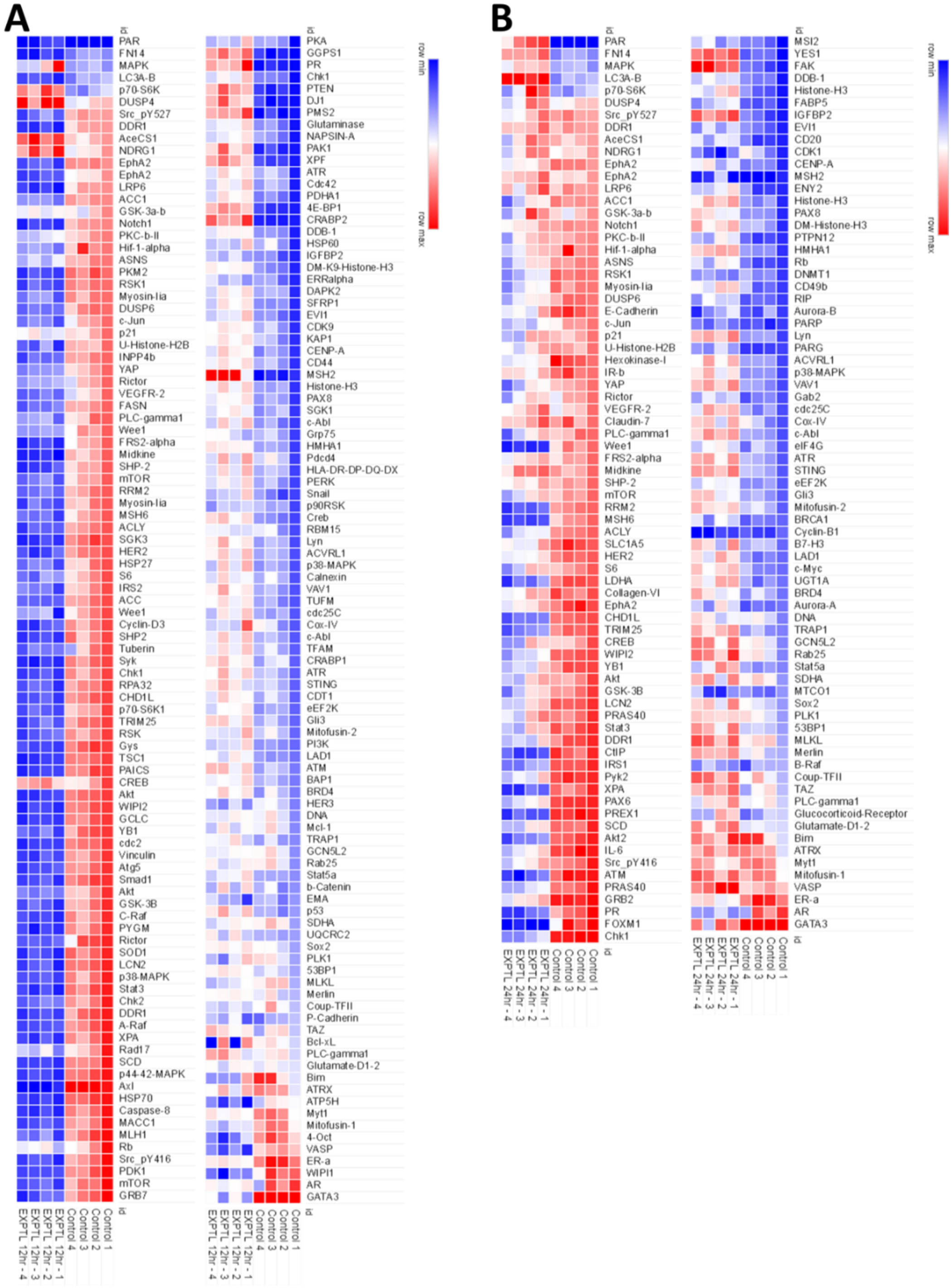

Supplementary Figure 8: Heat map of RPPA analysis for phosphorylation changes with P<0.05 at 12h (**A**) and 24h (**B**). *Statistical analysis was done using a two-tailed Student's t test to compare the means of protein/phosphorylation expression between control samples and metixene treated samples.*

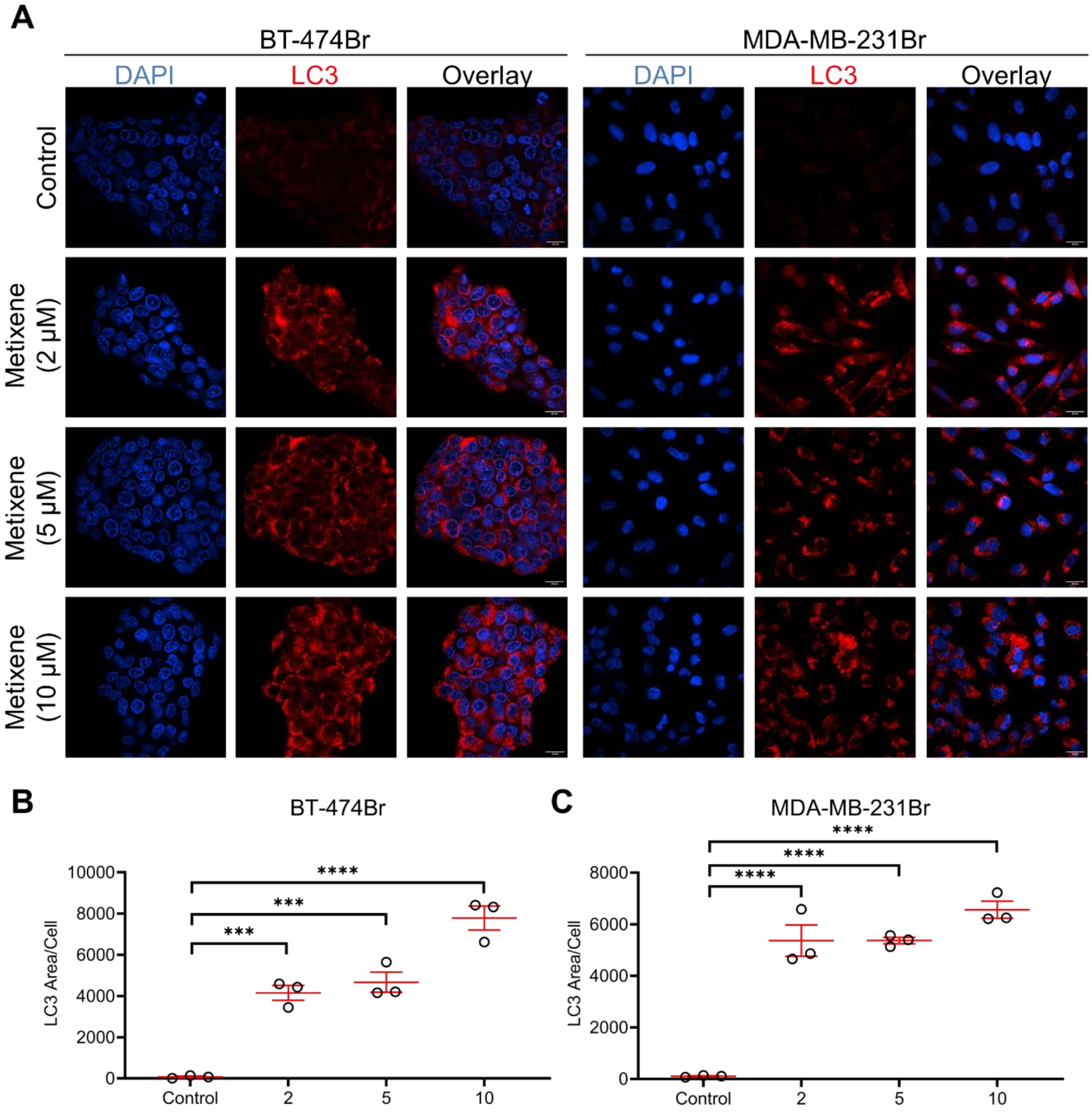

Supplementary Figure 9: Immunofluorescence staining of LC3 in metixene-treated cells in a dose-dependent manner. **A**, Representative LC3 puncta immunofluorescence in BT-474Br and MDA-MB-231Br cells under metixene in a dose-dependent manner. **B**, Quantification of LC3 area per cell in BT-474Br cells. **C**, Quantification of LC3 area per cell in MDA-MB-231Br cells. *The results are representative of three independent experiments. Statistical analysis was performed using one-way ANOVA with post hoc Dunnett's test. ****$p<0.0001$; ***$p<0.001$; **$p<0.01$; *$p<0.05$*

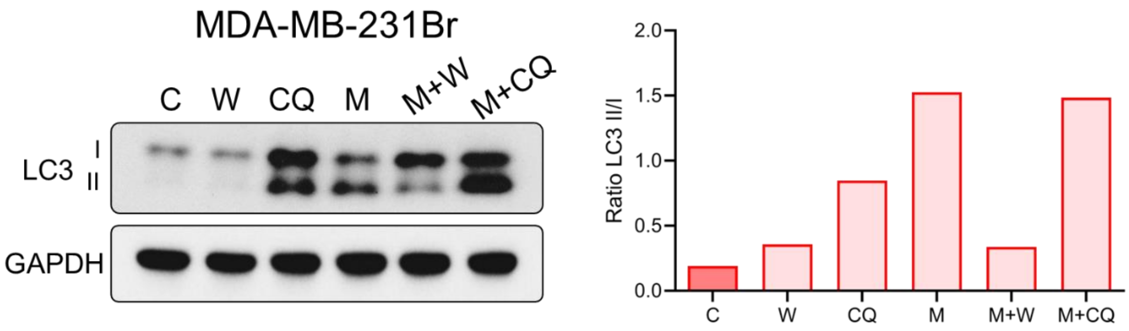

Supplementary Figure 10: Autophagy flux induction using metixene. Comparison of LC3 expression in cells treated with control (C), wortmannin (W, 20 nM), chloroquine (CQ, 20 µM), Metixene (M, 10 µM), M+W, and M+CQ for 24 h.

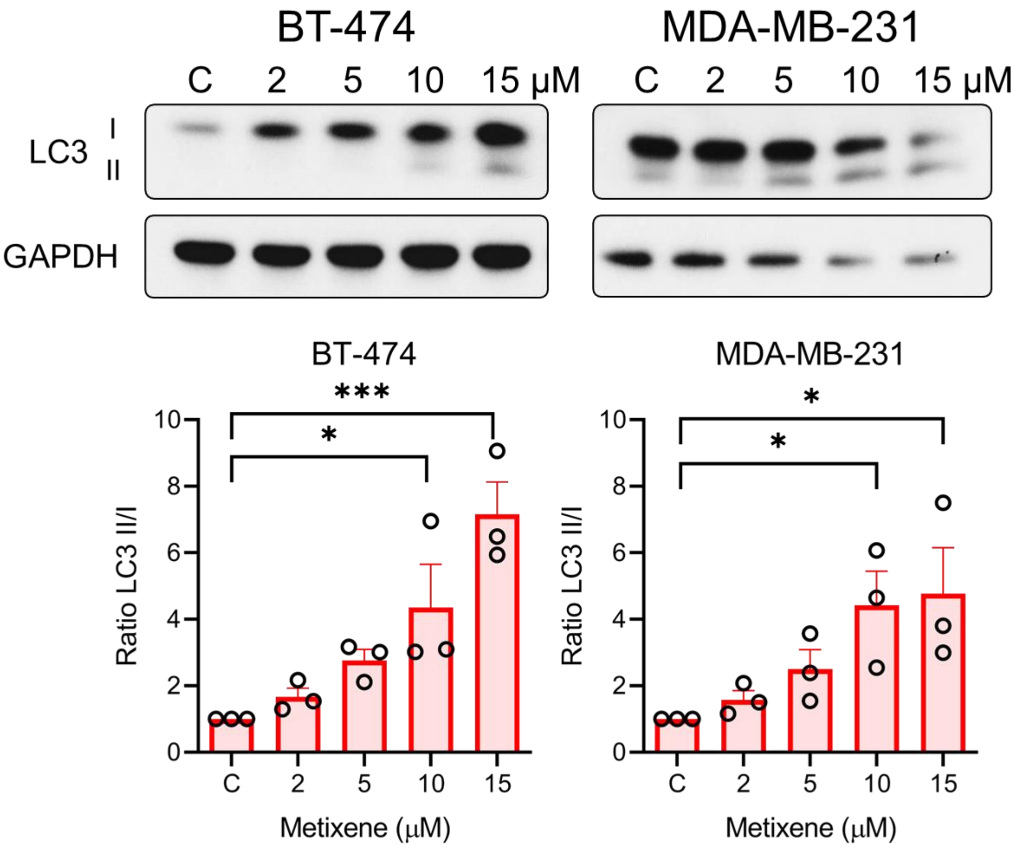

Supplementary Figure 11: Western blots showing LC3II/I expression in primary breast cancer cell lines. *The results are representative of three independent experiments, and bar graphs represent means ± SEM. Statistical analysis was done using the Student's t test. ****p<0.0001; ***p<0.001; **p<0.01; *p<0.05*

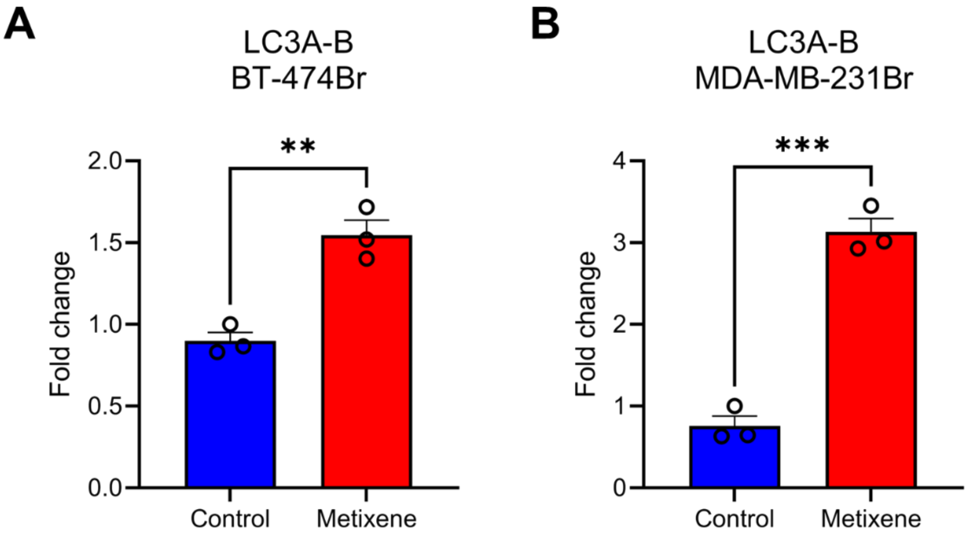

Supplementary Figure 12: Quantification of the area of LC3A-B IHC staining in the metastatic brain tumors of metixene-treated mice vs. controls after (**A**) intracranial injection of HER2-positive BT-474Br cells and (**B**) intracarotid injection of triple negative MDA-MB-231Br cells *in vivo. The results are representative of three independent experiments, and bar graphs represent means ± SEM. Statistical analysis was done using the Student's t test. ****p<0.0001; ***p<0.001; **p<0.01; *p<0.05*

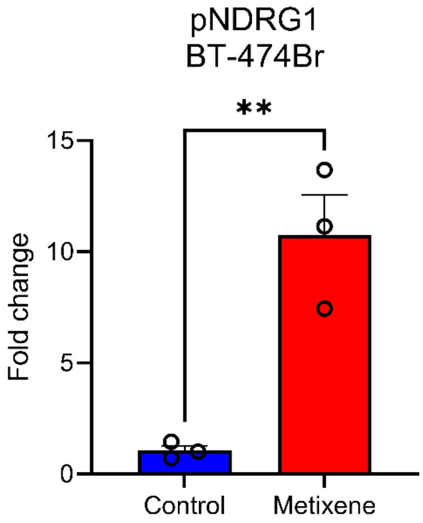

Supplementary Figure 13: Quantification of the area of pNDRG1 IHC staining in the metastatic brain tumors of metixene-treated mice vs. controls after intracranial injection of HER2-positive BT-474Br cells *in vivo*. *The results are representative of three independent experiments, and bar graphs represent means ± SEM. Statistical analysis was done using the Student's t test. ****p<0.0001; ***p<0.001; **p<0.01; *p<0.05*

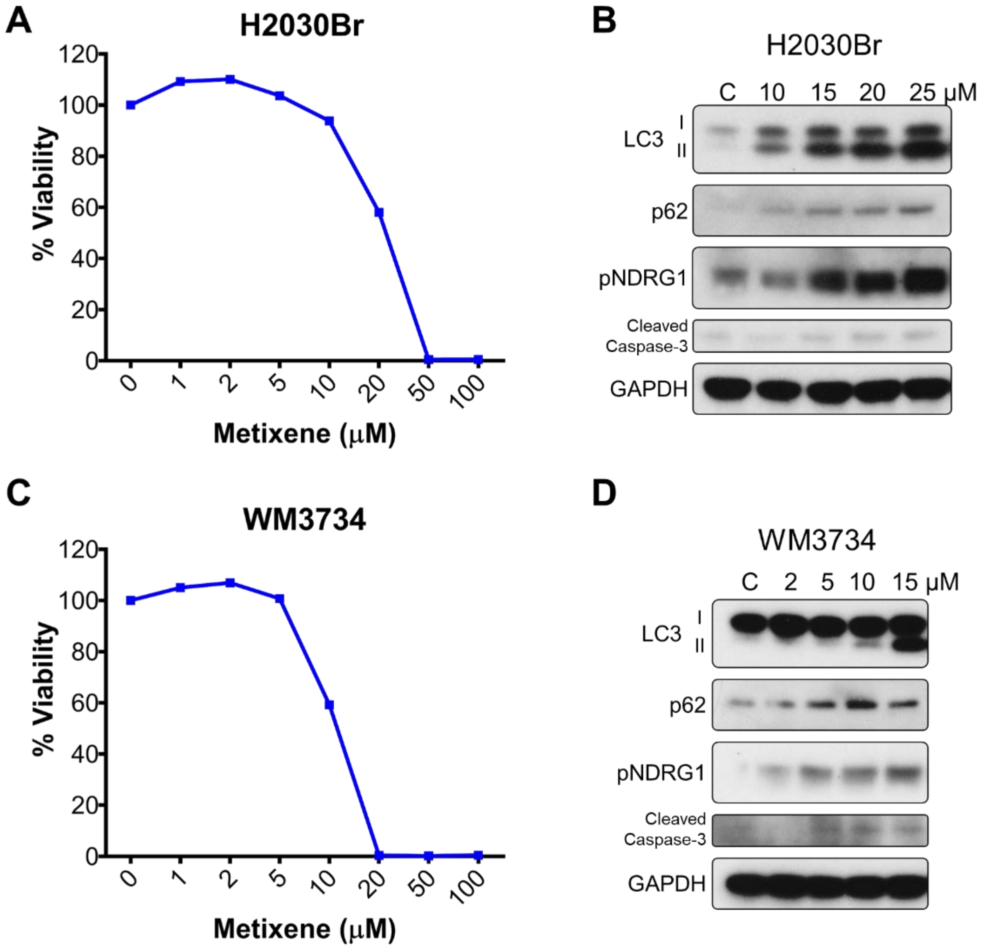

Supplementary Figure 14: Metixene induces incomplete autophagy and caspase-mediated apoptosis in metastatic brain cancer of different primary origins. **A**, Cell viability of a human lung cancer brain metastases cell line, H2030Br, at different concentrations of metixene after three days of treatment. **B**, Protein expression of LC3, p62, pNDRG1, and cleaved caspase-3 upon metixene treatment in H2030Br. **C**, Cell viability of a human melanoma brain metastases cell line, WM3734, at different concentrations of metixene after three days of treatment. **D**, Protein expression of LC3, p62, pNDRG1, and cleaved caspase-3 upon metixene treatment in WM3734.

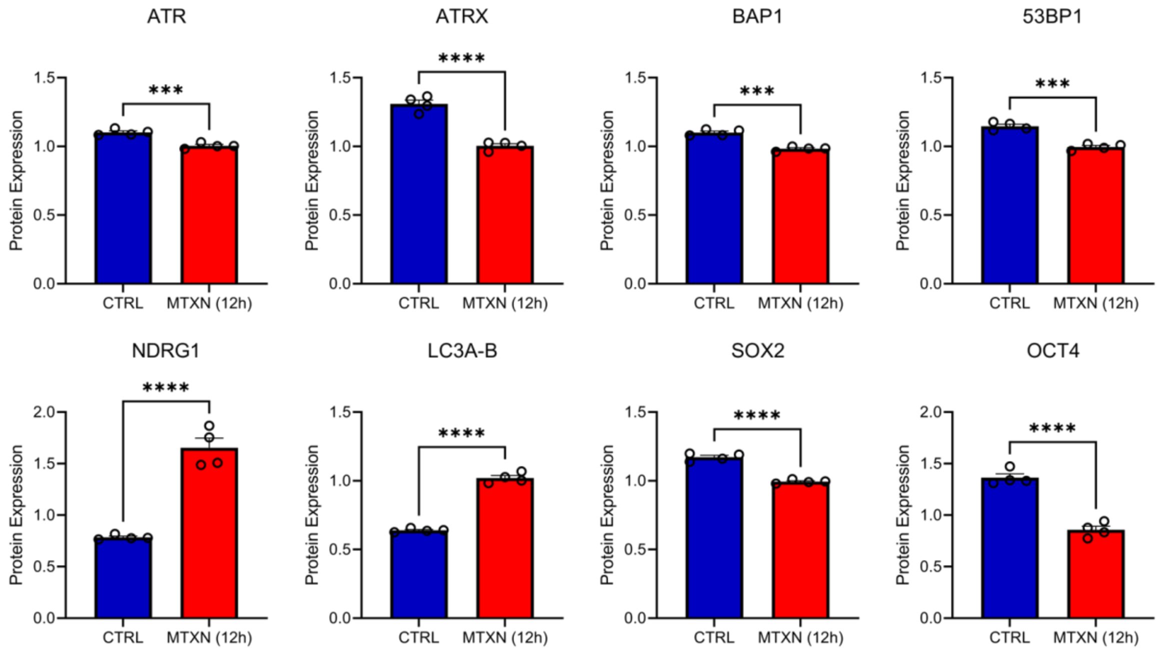

Supplementary Figure 15: Protein expression of DNA damage repair proteins (ATR, ATRX, BAP1, and 53BP1), cellular stress marker (NDRG1), autophagy marker (LC3A-B), and stemness markers (SOX2 and OCT4) after 12 hours of metixene treatment in BT-474Br cells. *The results are representative of four technical replicates, and bar graphs represent means ± SEM. Statistical analysis was done using the Student's t test.* ****$p<0.0001$; ***$p<0.001$; **$p<0.01$; *$p<0.05$

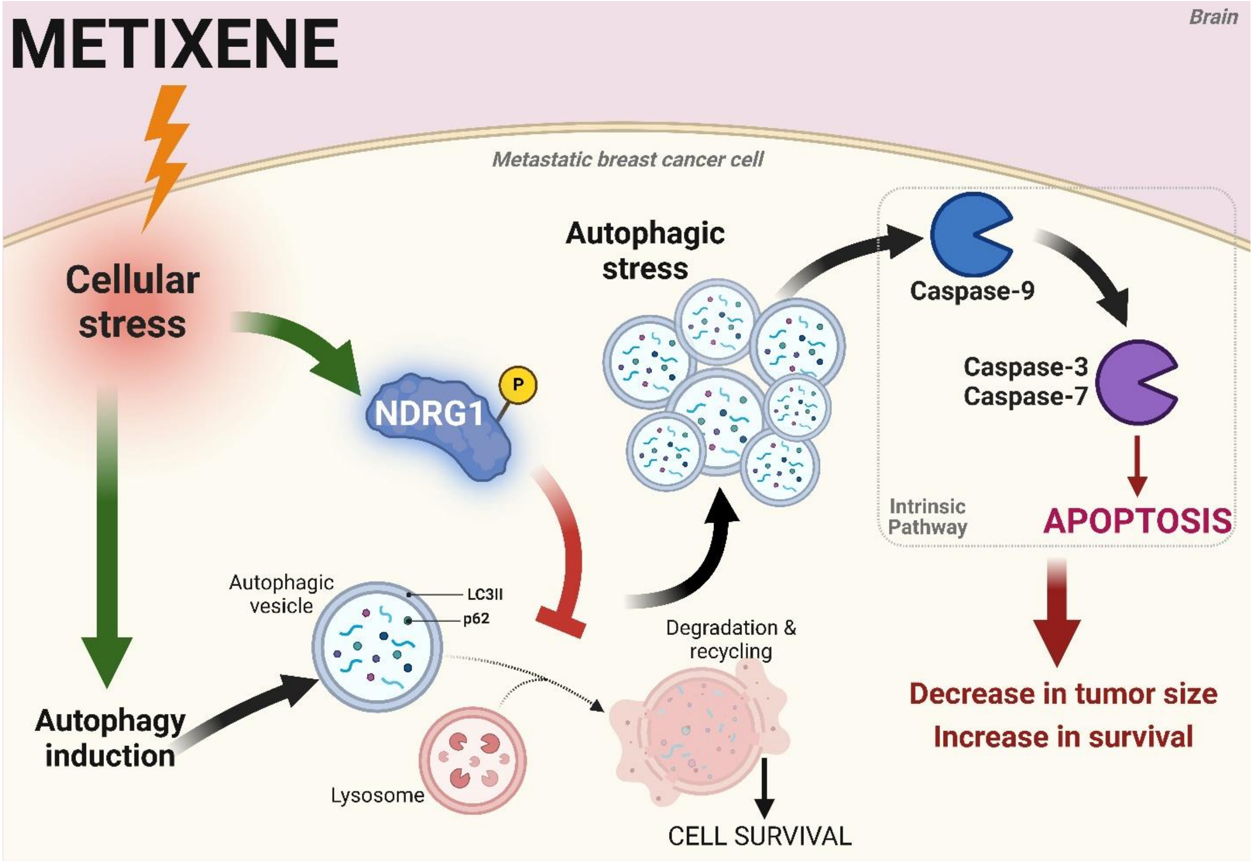

Supplementary Figure 16: A schematic illustration showing the proposed mechanism of action of metixene, through incomplete autophagy, in metastatic brain cancer cells.

Figure 5A

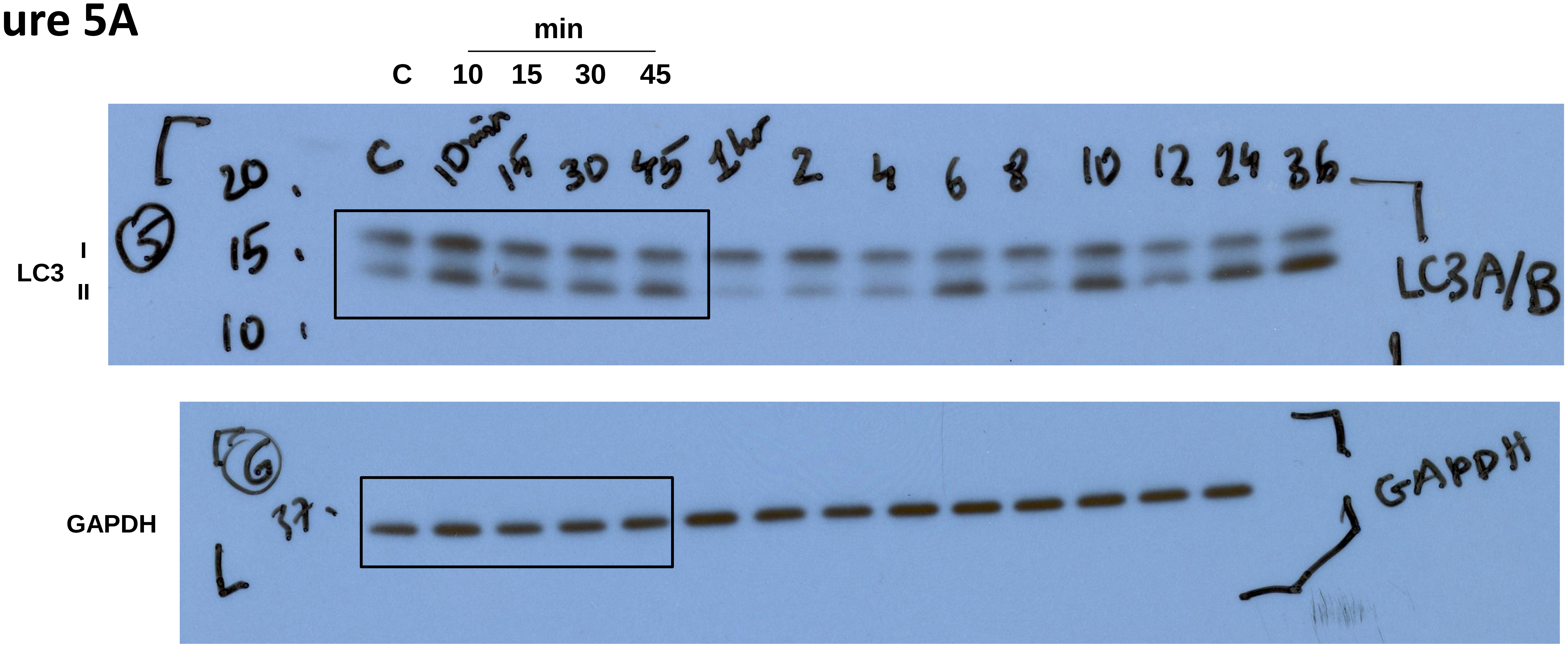

Figure 5B

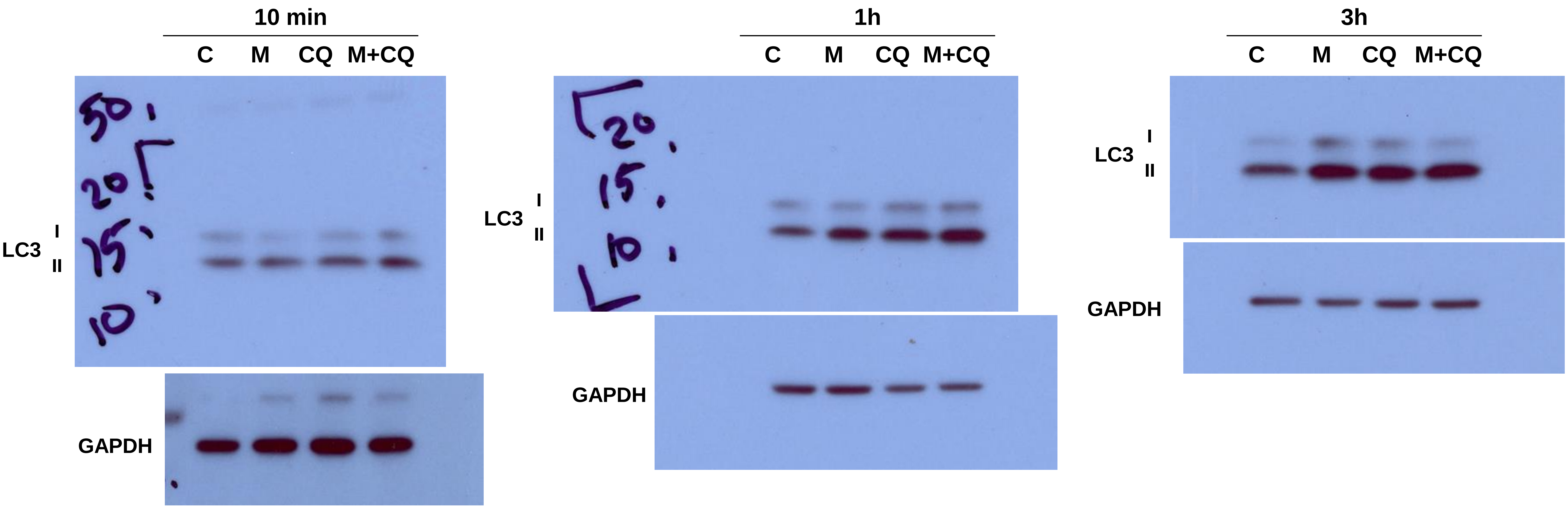

Figure 6C

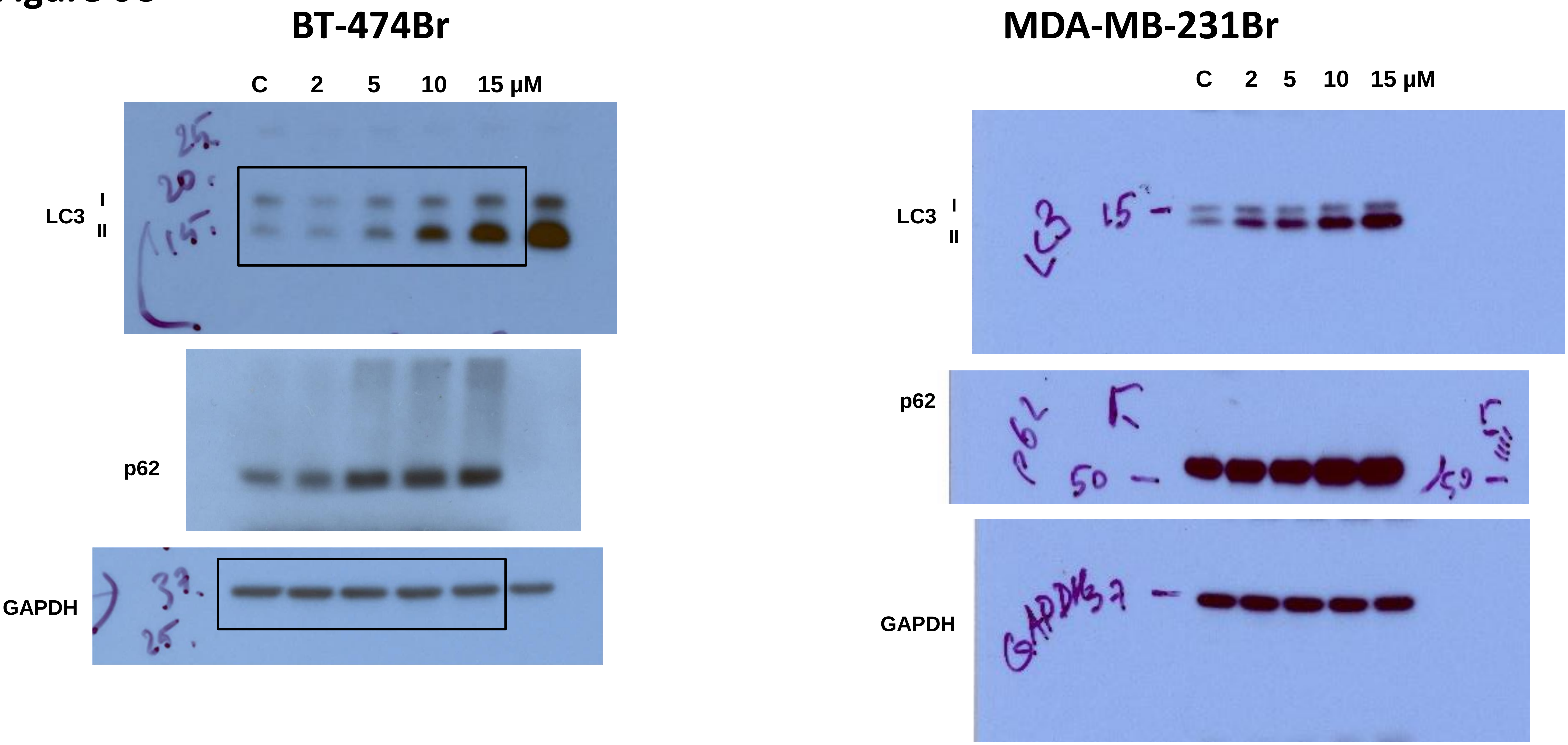
BT-474Br
C 2 5 10 15 µM
LC3
I
II
p62
GAPDH
MDA-MB-231Br
C 2 5 10 15 µM
LC3
I
II
p62
GAPDH

Figure 7A

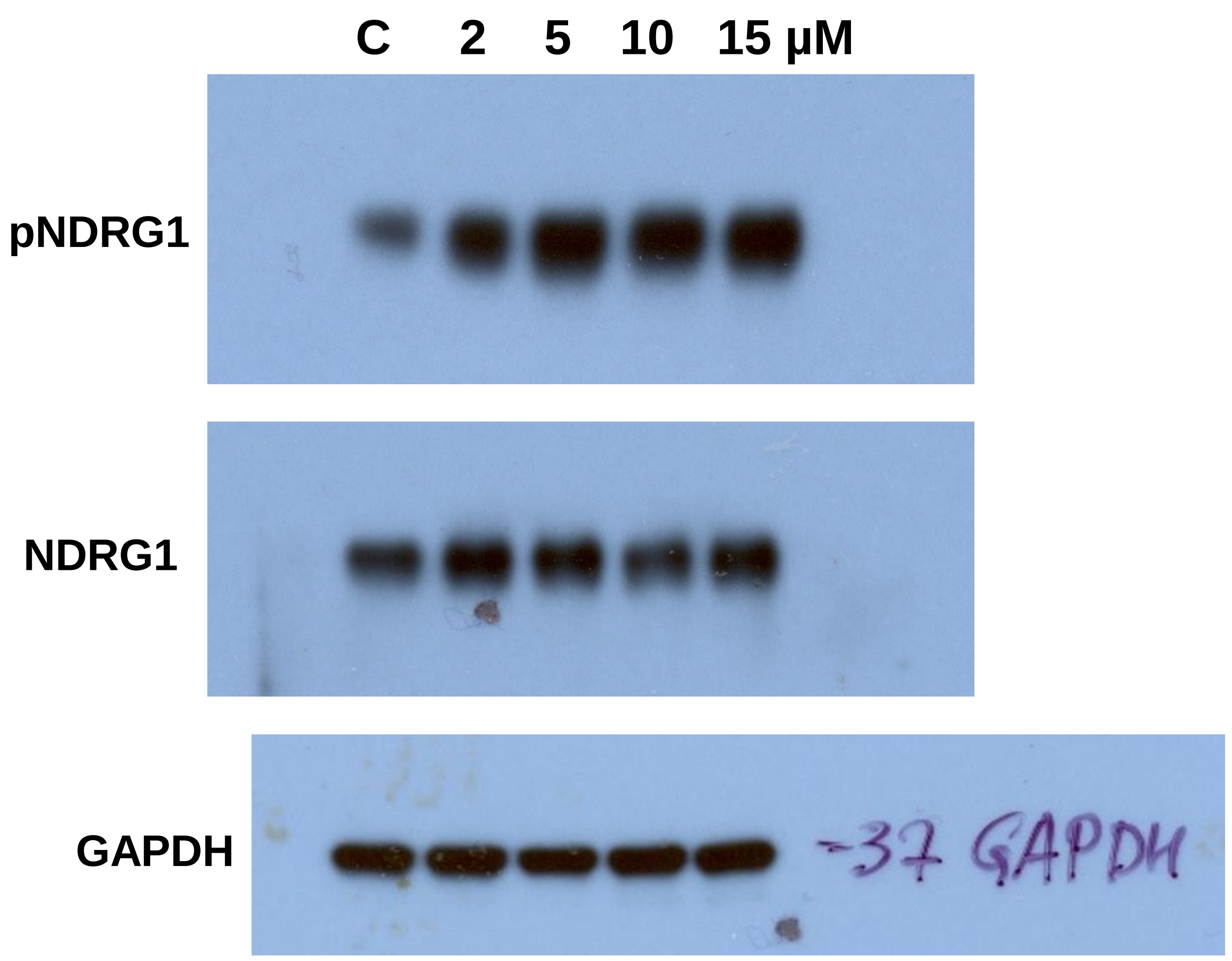

Figure 7B

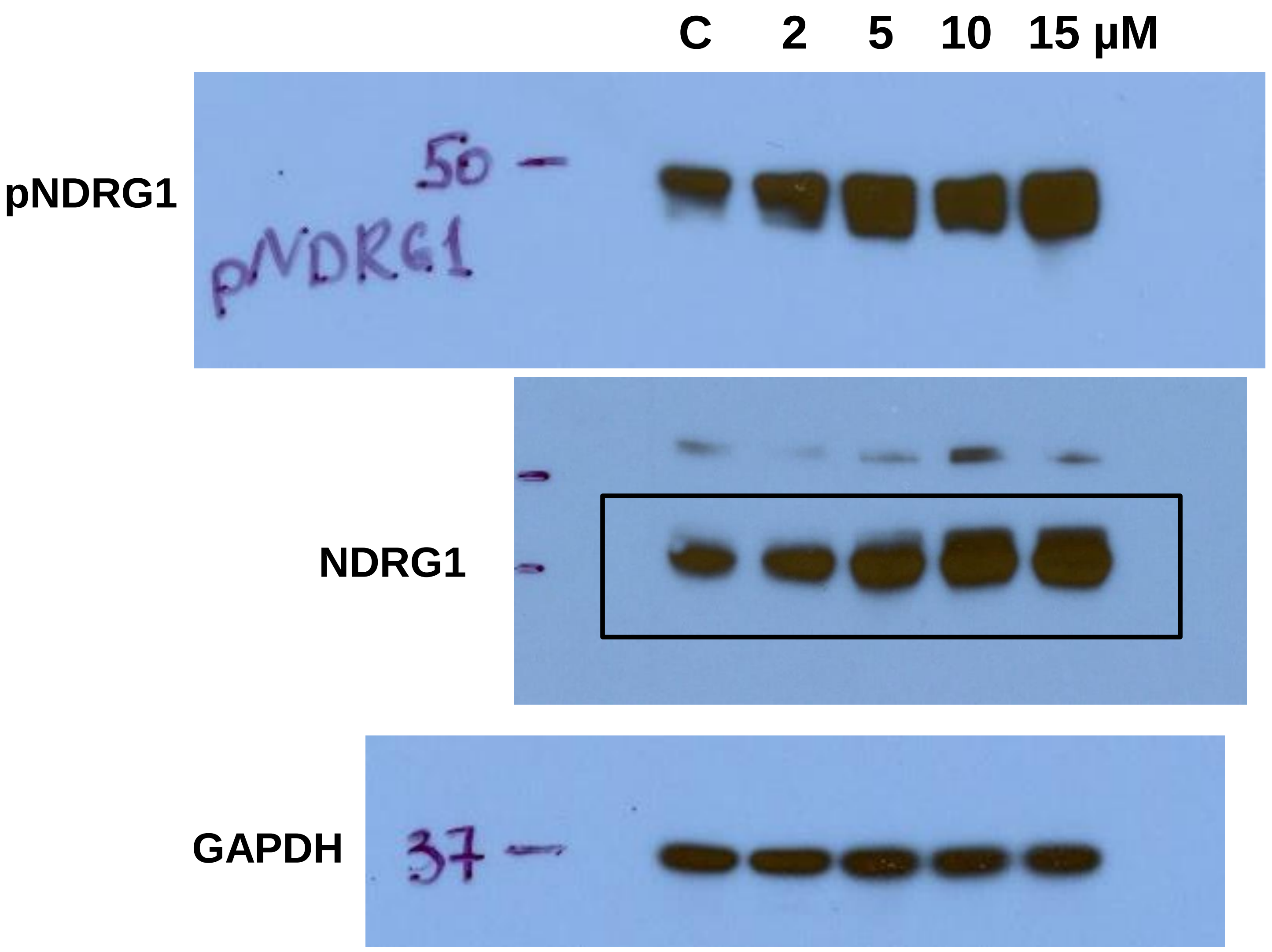

Figure 7C

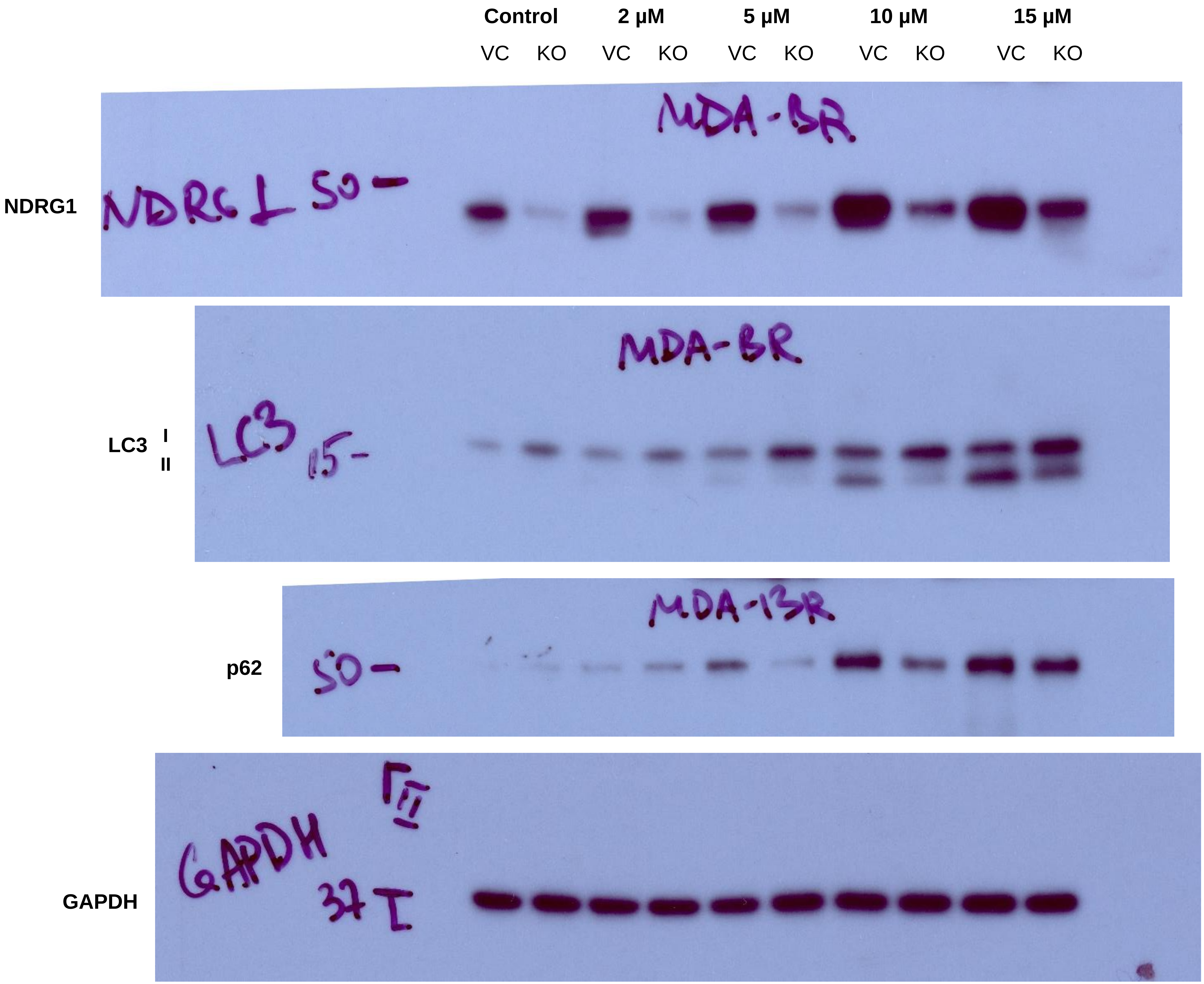

Control
2 μM
5 μM
10 μM
15 μM
VC KO VC KO VC KO VC KO VC KO
NDRG1
LC3 I II
p62
GAPDH
MDA-BR
50-
15-
37

Figure 7F

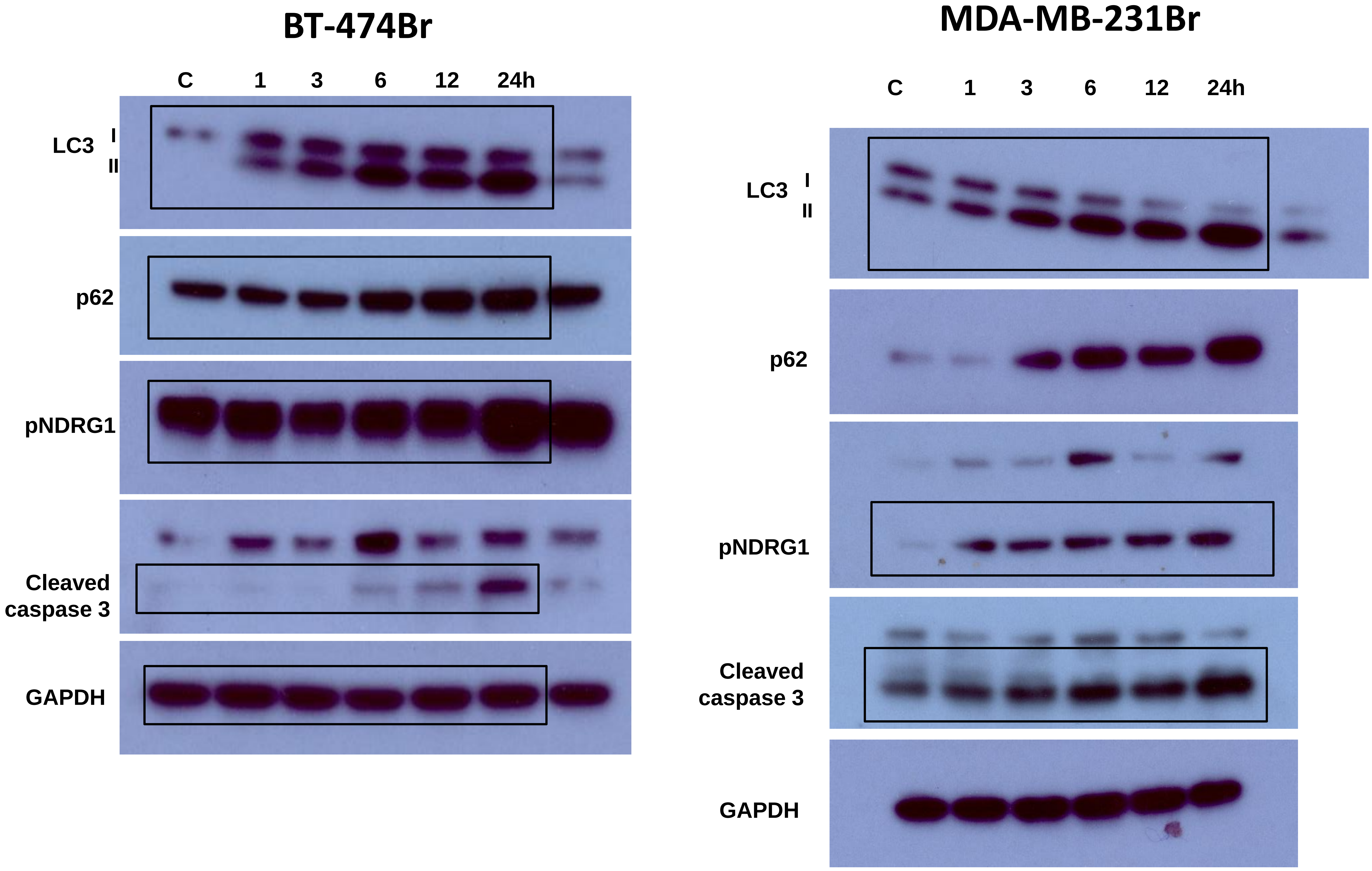
BT-474Br
C
1
3
6
12
24h
LC3
I
II
p62
pNDRG1
Cleaved caspase 3
GAPDH
MDA-MB-231Br
C
1
3
6
12
24h
LC3
I
II
p62
pNDRG1
Cleaved caspase 3
GAPDH

Supplementary Figure 10

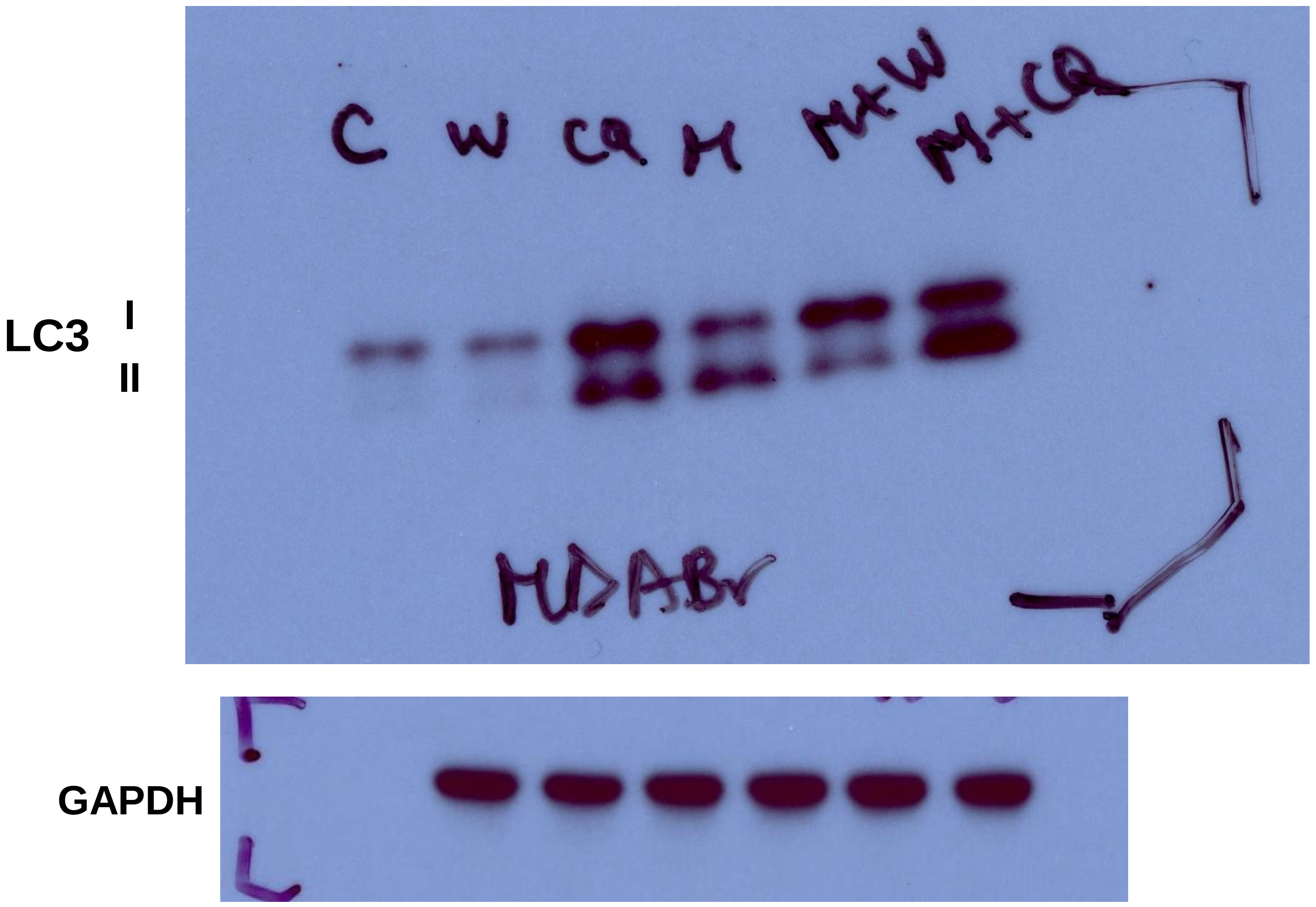

Supplementary Figure 11

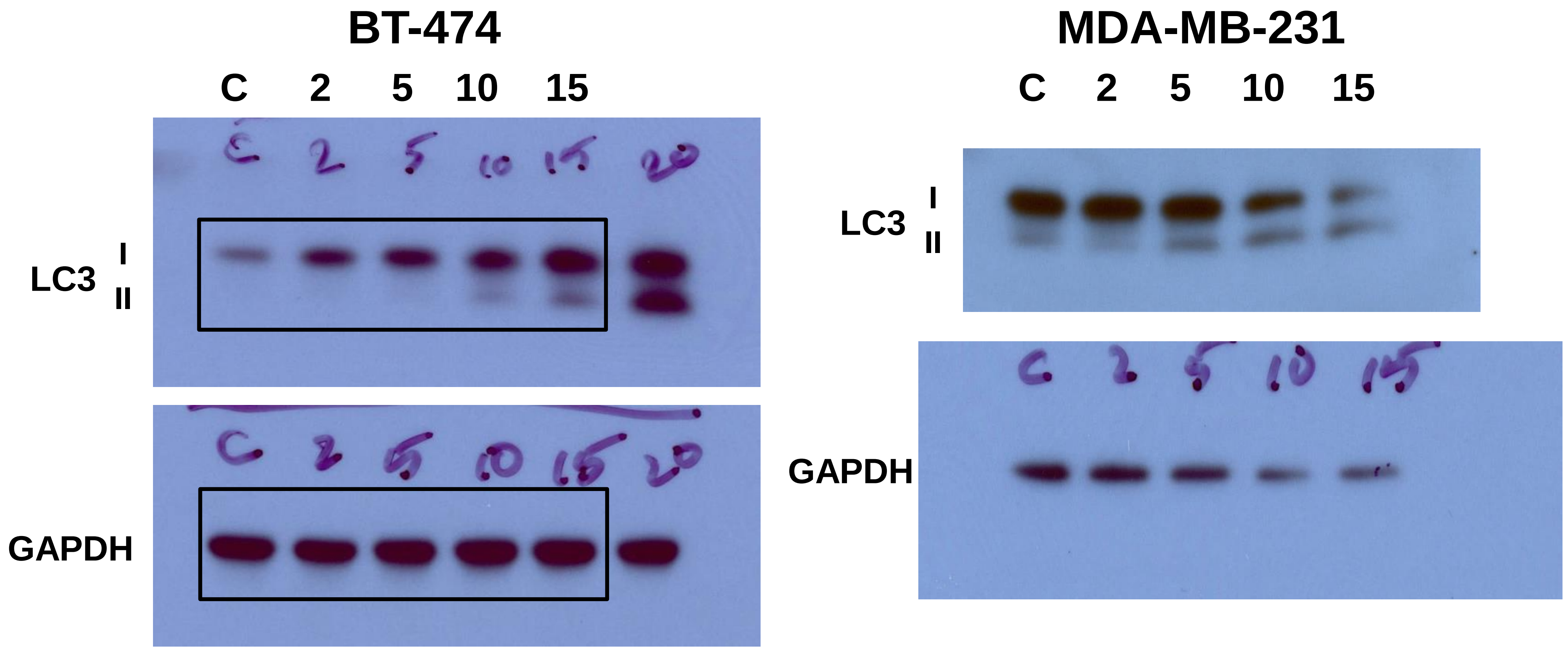

Supplementary Figure 14B

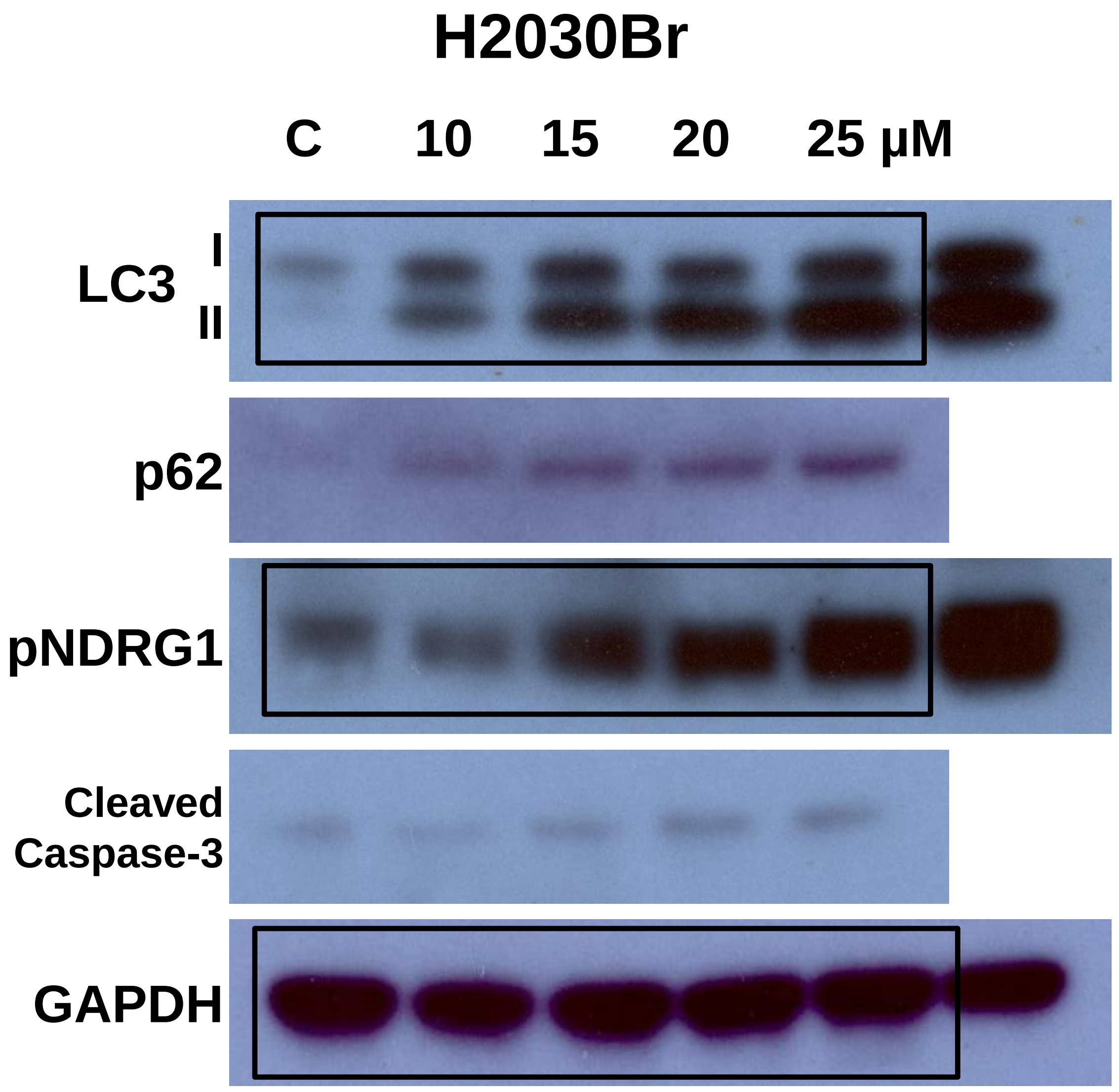

Supplementary Figure 14D

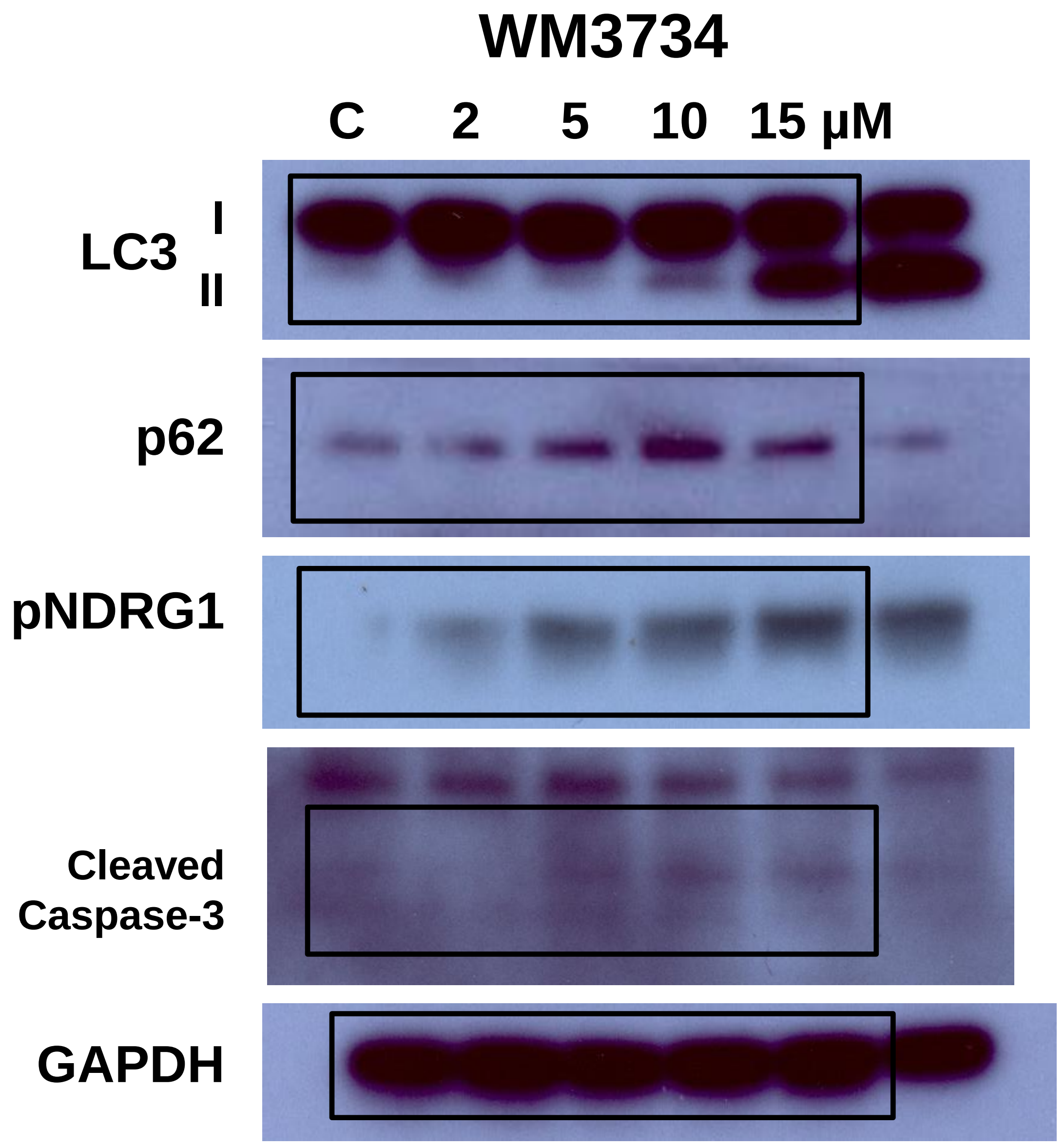

Supplement: Supplemental data [file jci-133-161142-s052.pdf]
